# RNase L Amplifies Interferon Signaling by Inducing Protein Kinase R-Mediated Antiviral Stress Granules

**DOI:** 10.1128/JVI.00205-20

**Published:** 2020-06-16

**Authors:** Praveen Manivannan, Mohammad Adnan Siddiqui, Krishnamurthy Malathi

**Affiliations:** a Department of Biological Sciences, University of Toledo, Toledo, Ohio, USA; b Department of Medicine, Division of Infectious Diseases, Columbia University Medical Center, New York, New York, USA; Hudson Institute of Medical Research

**Keywords:** PKR, RNase L, Rig-I, double-stranded RNA, interferon, stress granules

## Abstract

Virus infection leads to activation of the interferon (IFN)-induced endoribonuclease RNase L, which results in degradation of viral and cellular RNAs. Both cellular and viral RNA cleavage products of RNase L bind pattern recognition receptors (PRRs), like retinoic acid-inducible I (Rig-I) and melanoma differentiation-associated protein 5 (MDA5), to further amplify IFN production and antiviral response. Although much is known about the mechanics of ligand binding and PRR activation, how cells coordinate RNA sensing with signaling response and interferon production remains unclear. We show that RNA cleavage products of RNase L activity induce the formation of antiviral stress granules (avSGs) by regulating activation of double-stranded RNA (dsRNA)-dependent protein kinase R (PKR) and recruit the antiviral proteins Rig-I, PKR, OAS, and RNase L to avSGs. Biochemical analysis of purified avSGs showed interaction of a key stress granule protein, G3BP1, with only PKR and Rig-I and not with OAS or RNase L. AvSG assembly during RNase L activation is required for IRF3-mediated IFN production, but not IFN signaling or proinflammatory cytokine induction. Consequently, cells lacking avSG formation or RNase L signaling produced less IFN and showed higher susceptibility during Sendai virus infection, demonstrating the importance of avSGs in RNase L-mediated host defense. We propose a role during viral infection for RNase L-cleaved RNAs in inducing avSGs containing antiviral proteins to provide a platform for efficient interaction of RNA ligands with pattern recognition receptors to enhance IFN production to mount an effective antiviral response.

**IMPORTANCE** Double-stranded RNAs produced during viral infections serve as pathogen-associated molecular patterns (PAMPs) and bind pattern recognition receptors to stimulate IFN production. RNase L is an IFN-regulated endoribonuclease that is activated in virus-infected cells and cleaves single-stranded viral and cellular RNAs. The RNase L-cleaved dsRNAs signal to Rig-like helicases to amplify IFN production. This study identifies a novel role of antiviral stress granules induced by RNase L as an antiviral signaling hub to coordinate the RNA ligands with cognate receptors to mount an effective host response during viral infections.

## Abstract (precis)

Double-stranded RNAs produced during viral infections serve as pathogen-associated molecular patterns (PAMPs) and bind pattern recognition receptors to stimulate IFN production. RNase L is an IFN-regulated endoribonuclease that is activated in virus-infected cells and cleaves single-stranded viral and cellular RNAs. The RNase L-cleaved dsRNAs signal to Rig-like helicases to amplify IFN production. This study identifies a novel role of antiviral stress granules induced by RNase L as an antiviral signaling hub to coordinate the RNA ligands with cognate receptors to mount an effective host response during viral infections.

## INTRODUCTION

Viral invasion and replication are detected in host cells by pathogen recognition receptors (PRRs), triggering signaling pathways that result in production of type 1 interferon (IFN) (1–3). IFN produced by virus-infected cells acts in autocrine and paracrine ways by binding to cell surface receptors (IFN-α/β receptor [IFNAR]) to induce expression of antiviral IFN-stimulated genes (ISGs), including retinoic acid-inducible I (Rig-I), melanoma differentiation-associated protein 5 (MDA5), 2′-5′-oligoadenylate synthetase (OAS), RNase L, double-stranded RNA (dsRNA)-dependent protein kinase R (PKR), and interferon-induced proteins with tetratricopeptide repeats (IFIT), to perpetuate antiviral signaling (3, 4). Recognition of viral nucleic acids that serve as pathogen-associated molecular patterns (PAMPs) is accomplished by PRRs, like the endosomal Toll-like receptors (TLR3, -7/8, and -9), cytosolic Rig-I like receptors (RLRs) (Rig-I and MDA5), DExD/H-box helicases (DDX1, DDX21, DHX33, and DHX36), PKR, 2′,5′-OAS, and cytosolic DNA sensors (DAI, STING, and cGAS), by virtue of their compartment-specific distribution in cells and modification on the RNAs or DNA (5–12). RLRs detect cytoplasmic viral RNAs and discriminate self from viral RNAs by recognizing double-stranded structures and 5′-triphosphate, which are lacking in self RNAs (13). Rig-I and MDA5 contain an RNA helicase domain for binding RNA and a caspase recruitment domain (CARD) for downstream signaling (14). In the case of Rig-I, RNA binding allows K63 ubiquitination of the CARD by TRIM25, and ATPase activity induces conformational change and oligomerization (15). In contrast, Rig-I undergoes degradation after conjugation to the E3 ubiquitin ligase RNF125 (16). The Rig-I CARD interacts with the CARD-like domain of IFN-β promoter stimulator 1 (IPS-1/MAVS/VISA/Cardif) at the outer mitochondrial membrane via CARD-CARD interaction, which further activates TRAF3 and TBK1 (17–20). TBK1 phosphorylates IRF3, which translocates to the nucleus to induce IFN production (21, 22).

Double-stranded-RNA-dependent PKR is activated by binding dsRNA ligands and participates in integrated stress response during viral infections (23). RNA binding induces dimerization and autophosphorylation, resulting in activation and phosphorylation of eIF2α (eukaryotic initiation factor 2 alpha subunit) (24). Phosphorylated eIF2α (P-eIF2α) represses translation and causes aggregation of stalled translation preinitiation complexes containing mRNAs, initiation factors, small ribosomal subunits, and RNA-binding proteins, together with the Ras-GAP SH3 domain binding protein (G3BP) and T-cell-restricted intracellular antigen 1 (TIA 1), into stress granules (SGs) (25).

SGs are nonmembranous RNA-protein complexes that are formed in the cytoplasm in response to diverse stress signals, including viral infections (26). Complex RNA-protein interactions in the SGs establish a liquid-liquid phase separation from the rest of the cytoplasm, facilitating recruitment of multiple proteins into a dynamic compartment (27). Depending on the nature of the stress signal, protein kinases, such as PKR, GCN2 (general control nonderepressible 2), HRI (heme-regulated inhibitor), or PERK (PKR-like ER kinase), phosphorylate a translation initiation factor, eIF2α, to inhibit translation, which in turn promotes SG formation (28–30). The SG composition and proteins recruited vary depending on the type of stimulus and the cell type, but G3BP1 is required for nucleation in all contexts. SGs have been considered general triage sites for mRNA turnover, but recent studies show selective exclusion from SGs of some transcripts needed to overcome stress (31–33). Unlike the canonical SGs formed under stress conditions, antiviral SGs (avSGs) form during viral infections and have been proposed to play a role in antiviral signaling (34) by recruiting antiviral proteins, including PKR, Rig-I, MDA5, OAS, RNase L, Trim5, ADAR1, ZAP, cGAS, and RNA helicases, like DHX36, DDX3, and DDX6 (35–38). Assembly of avSGs is required for signaling to produce IFN during Newcastle disease virus (NDV), influenza A virus (IAV), and Sindbis virus (SINV) infections. During IAV infection, SGs are induced, and both IAV RNA and Rig-I are sequestered in SGs, thereby providing a platform for sensing of viral RNA by Rig-I (35, 39). In addition, antiviral proteins, like PKR, OAS, RNase L, LGP2, and MDA5, were also shown to coalesce in avSGs (35, 36, 40, 41). Several viruses counteract SG formation by targeting SG proteins through G3BP1 cleavage or by inhibiting the upstream eIF2α pathway to support viral replication, suggesting an important role of SGs in viral pathogenesis (42, 43).

In most vertebrates, viruses induce an RNA degradation process that is regulated through the action of the ubiquitous cellular endoribonuclease RNase L. Type I IFN produced during viral infections transcriptionally induces OAS proteins that are activated by binding dsRNA to produce a unique 2′,5′-oligoadenylate, 2-5A [p*x*5′A(2′p5′A)*n*; *x* = 1 to 3; *n* ≥ 2], produced from cellular ATP. The only established function of 2-5A is activation of RNase L. RNase L is expressed as an inactive monomer, and binding 2-5A promotes dimerization and conversion to an active enzyme that targets single-stranded viral and cellular RNAs after UU/UA nucleotide sequences, resulting in dsRNA cleavage products with 5′-hydroxyl and 2′,3′-cyclic phosphate ends (10, 44, 45). While activity of RNase L on the viral genome or mRNA directly eliminates viruses, the dsRNA cleavage products signal through Rig-I/MDA5/MAVS (IPS-1) and IRF3 to induce IFN production (46). RNase L-cleaved RNAs also induce NLRP3 inflammasomes and promote a switch from RNase L-induced autophagy to apoptosis by promoting cleavage of the autophagy protein Beclin-1 (47, 48). The role of RNase L in generating dsRNA with IFN-inducing abilities and the multiple overlapping signaling pathways activated by avSGs during viral infections prompted us to explore the role of RNase L-cleaved RNAs in inducing avSG formation as a platform for antiviral signaling. Our results show that direct activation of RNase L with 2-5A or treatment with RNase L-cleaved RNAs induces avSG formation by activating PKR and phosphorylation of eIF2α. Characterization of purified avSGs showed the interaction of G3BP1 in avSGs with PKR and Rig-I, but not OAS or RNase L. AvSG assembly was required for IRF3-mediated IFN production, but not IFN signaling or proinflammatory cytokine induction, and affected viral pathogenesis. These studies demonstrate avSG assembly induced by RNase L to be an antiviral signaling hub to coordinate RNA ligands with PRRs to mount an effective antiviral response.

## RESULTS

### RNase L activation induces formation of antiviral stress granules containing antiviral proteins.

**FIG 1:**
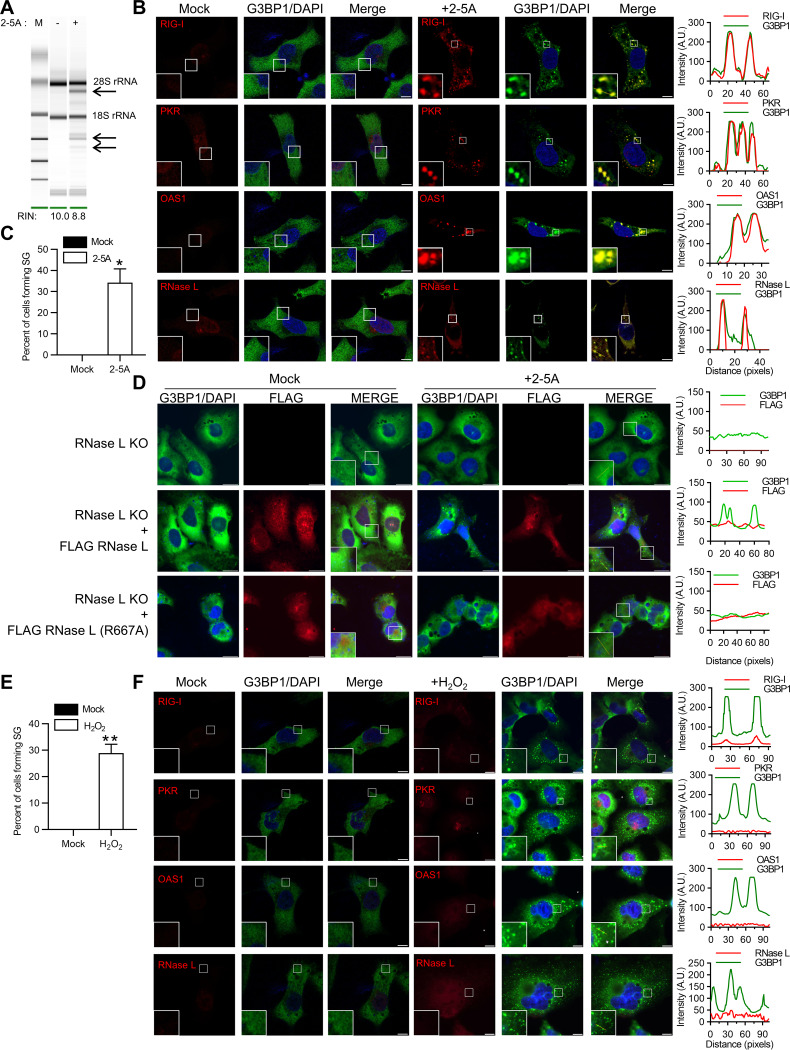
Activation of RNase L induces antiviral stress granule formation. (A) HT1080 cells were transfected with 2-5A (10 μM) for 8 h, and RNase L-mediated cleavage of rRNA (arrows) was analyzed on RNA chips using the Agilent Bioanalyzer 2100; RIN, RNA integrity number. (B) Cells were fixed and stained with G3BP1 and the indicated antiviral proteins. The magnified images correspond to the boxed regions. (Right) Intensity profiles of G3BP1 and antiviral proteins along the plotted lines, as analyzed by Image J line scan analysis. (C) The percentages of cells forming stress granules were quantitated. (D) RNase L KO cells were either mock transfected or transfected with FLAG-WT-RNase L or FLAG-R667A-RNase L and immunostained for G3BP1 and FLAG. (Right) Intensity profiles of G3BP1 and FLAG along the plotted lines as analyzed by Image J line scan analysis. (E) HT1080 cells were treated with H_2_O_2_ (1 mM) for 3 h, and the percentages of cells forming stress granules were quantitated. (F) The cells were immunostained with G3BP1 and the indicated antiviral proteins. (Right) Intensity profiles of G3BP1 and antiviral proteins along the plotted lines as analyzed by Image J line scan analysis. All the experiments included at least 100 cells from three replicates. Scale bars, 10 μm. The data are representative of at least three independent experiments. *, *P* < 0.01; **, *P* < 0.001. A.U., arbitrary units.

Viral infection or dsRNA causes aggregation of Rig-I, PKR, OAS, and RNase L in avSGs for production of type I IFN by providing a platform for integrating RNA ligands with antiviral proteins. The role of dsRNA by-products of RNase L enzyme activity in regulating type I IFN production prompted us to explore avSG formation during RNase L activation. Transfection of HT1080 fibrosarcoma cells with 2-5A, a highly specific ligand and activator of RNase L, resulted in a characteristic rRNA cleavage pattern (Fig. 1A) and localization of a key stress granule protein, G3BP1, in distinct stress granules compared to diffuse distribution in mock-treated cells (Fig. 1B). To determine if these 2-5A-induced stress granules were antiviral stress granules, we performed immunofluorescence assays for the RNA-binding antiviral proteins Rig-I, PKR, OAS, and RNase L with G3BP1. We observed significant colocalization of these antiviral proteins on RNase L activation in avSGs (Fig. 1B). Following 2-5A transfection, compared to mock-treated cells, 32% of cells formed stress granules (Fig. 1C). To demonstrate that avSGs were formed in response to RNase L activation, we generated CRISPR-mediated knockout of RNase L in HT1080 cells (49) and observed no avSG formation with 2-5A transfection (Fig. 1D). AvSG formation was restored in these cells only by expression of Flag-wild-type (WT) RNase L and not a Flag-RNase L R667A mutant that lacked enzyme activity (Fig. 1D) (50). These results suggest that direct activation of RNase L by 2-5A induces the formation of avSGs and that Rig-I, PKR, OAS, and RNase L are recruited to these avSGs.

Antiviral stress granules are distinct from canonical stress granules and are characterized by the presence of antiviral RNA-binding proteins, RNA helicases, and RNA ligands that form during viral infection (51). To demonstrate the distinct nature of avSGs, we treated HT1080 cells with H_2_O_2_ to induce oxidative stress, which promotes the formation of stress granules. While SGs formed in 30% of the H_2_O_2_-treated cells, as shown by G3BP1 puncta, there was no colocalization of the antiviral protein Rig-I, PKR, OAS, or RNase L in these granules (Fig. 1E and F). These results support observations made by others and demonstrate that avSGs are unique and distinct from canonical SGs that form in response to diverse stress stimuli, including oxidative stress (35, 36).

### RNase L-cleaved small RNAs activate PKR to induce avSGs.

**FIG 2:**
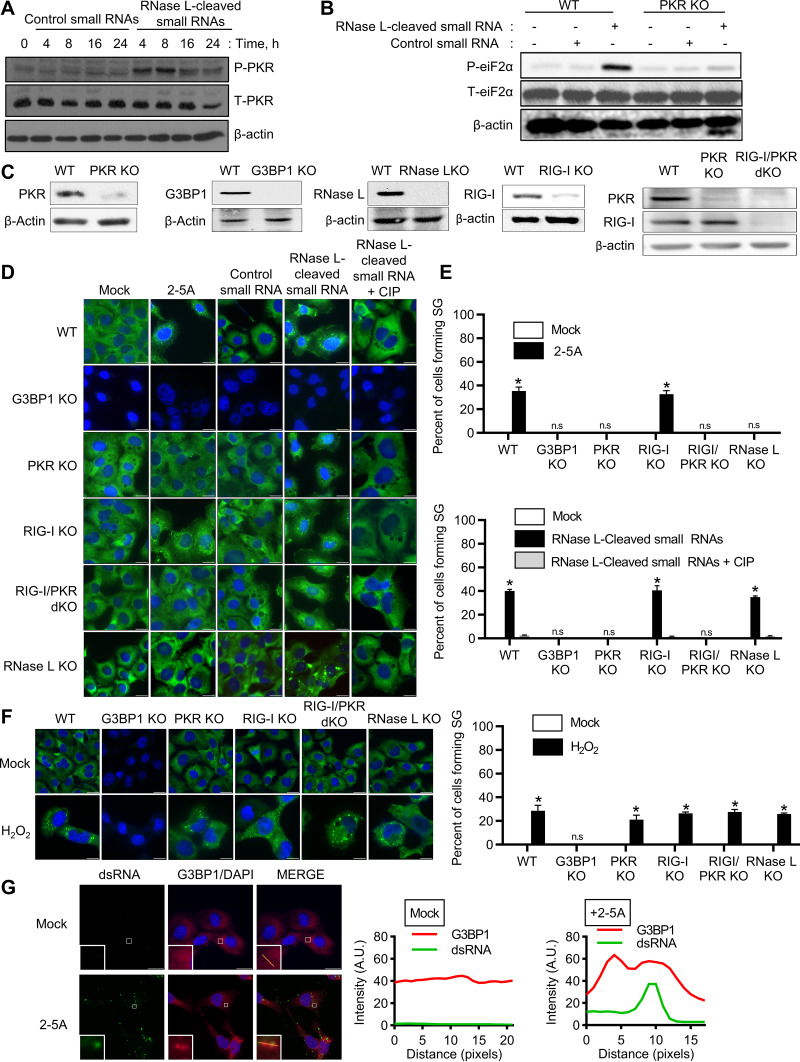
Involvement of PKR in RNase L-mediated avSG formation. (A) HT1080 cells were treated with RNase L-cleaved small RNAs or control small RNAs (2 μg/ml), and phosphorylation of PKR was detected in immunoblots. T-PKR, total PKR. (B) WT and PKR KO cells were treated with control small RNAs or RNase L-cleaved small RNAs (2 μg/ml) for 8 h, and phosphorylation of eIF2α levels was determined in immunoblots, (C) CRISPR/Cas9 knockout of G3BP1, RIG-I, PKR, RIG-I/PKR, or RNase L was verified in cell lysates by immunoblotting using specific antibodies. (D) The indicated cells were treated with 10 μM 2-5A, 2 μg/ml control small RNAs, 2 μg/ml RNase L-cleaved small RNAs, or 2 μg/ml calf intestinal alkaline phosphatase (CIP)-treated RNase L-cleaved small RNAs for 8 h. (E) The percentages of cells forming stress granules were quantitated. (F) The indicated cells were treated with 1 mM H_2_O_2_ for 3 h. Stress granule formation was analyzed by staining for G3BP1, and the percentages of cells forming stress granules were quantitated. (G) HT1080 cells were treated with 2-5A (10 μM) for 8 h, and cells were fixed and immunostained with G3BP1 and dsRNA; the magnified images correspond to the boxed regions. (Right) Intensity profiles of G3BP1 and dsRNA along the plotted lines as analyzed by Image J line scan analysis. All experiments included at least 100 cells from three replicates. The data are representative of three independent experiments. Scale bars, 20 μm. *, *P* < 0.01; n.s, not significant.

The RNA cleavage products of RNase L are predominantly small dsRNAs that signal via Rig-I and/or MDA5 and MAVS (IPS-1) to amplify IFN signaling (46). The stress-induced eIF2α kinases, like PKR, PERK, GCN2, and HRI, phosphorylate eIF2α, resulting in the formation of SGs. PKR is activated by binding dsRNA, so we tested the hypothesis that RNase L-cleaved small RNAs activate PKR and promote the formation of avSGs by phosphorylation of eIF2α. RNase L-cleaved small RNAs or control small RNAs were purified as previously described (46), and phosphorylation of PKR was monitored following transfection at the indicated times in HT1080 cells. RNase L-cleaved small RNAs induced autophosphorylation of PKR 4 h posttransfection that increased over time compared to control small RNAs (Fig. 2A). PKR phosphorylation was correlated with eIF2α phosphorylation only in cells treated with RNase L-cleaved small RNAs, but not control small RNAs (Fig. 2B). No phosphorylation of eIF2α by RNase L-cleaved RNAs was observed in cells lacking PKR generated by CRISPR-Cas9 technology (Fig. 2B and C). Taken together, these results indicate that RNase L-cleaved small RNAs activate PKR to phosphorylate eIF2α. To determine if PKR-induced phosphorylation of eIF2α translates into avSG formation, we analyzed avSG formation in PKR knockout (KO) cells by immunofluorescence analysis; compared it with G3BP1 KO, RNase L KO, Rig-I KO, and PKR/Rig-I double-KO (dKO) cells; and calculated the frequency of avSGs (Fig. 2D and E). As expected, about 35% of 2-5A-transfected WT and Rig-I KO cells formed avSGs, and cells lacking RNase L or PKR and PKR/Rig-I dKO cells did not form avSGs. G3BP1 is necessary to form avSGs in response to RNase L activation, as cells lacking G3BP1 did not induce avSGs. When the RNase L-cleaved RNAs were introduced into cells, in addition to WT (38%) and Rig-I KO (39%) cells, cells lacking RNase L also formed avSGs (34%), suggesting a role for the RNase L-cleaved RNA products in promoting avSG formation. Control small RNAs did not induce avSGs in any of the cells. Previous studies showed that the presence of 5′ OH and 2′,3-cyclic phosphoryl on RNase L-cleaved products contribute to IFN production, as removal of the 2′,3-cyclic phosphates by treatment with calf intestinal phosphatase (CIP) reduced IFN production (46). Removal of terminal 2′,3-cyclic phosphoryl on RNase L-cleaved RNAs significantly reduced avSG formation (Fig. 2D and E). In contrast, formation of canonical SGs in response to oxidative stress by H_2_O_2_ was not impacted in cells lacking PKR, RNase L, Rig-I, or both PKR and Rig-I (Fig. 2F). Finally, to demonstrate that dsRNA produced by RNase L activation with 2-5A localizes with G3BP1 in avSGs, 2-5A-transfected cells were stained with monoclonal antibody against dsRNA, and the highlighted regions showed colocalization with G3BP1 in avSGs in immunofluorescence assays (Fig. 2G). These findings demonstrate that RNase L enzyme activity produces small dsRNAs that activate PKR to phosphorylate eIF2α and induce formation of avSGs.

### Colocalization of PKR, Rig-I, OAS, and RNase L in avSGs upon RNase L activation.

**FIG 3:**
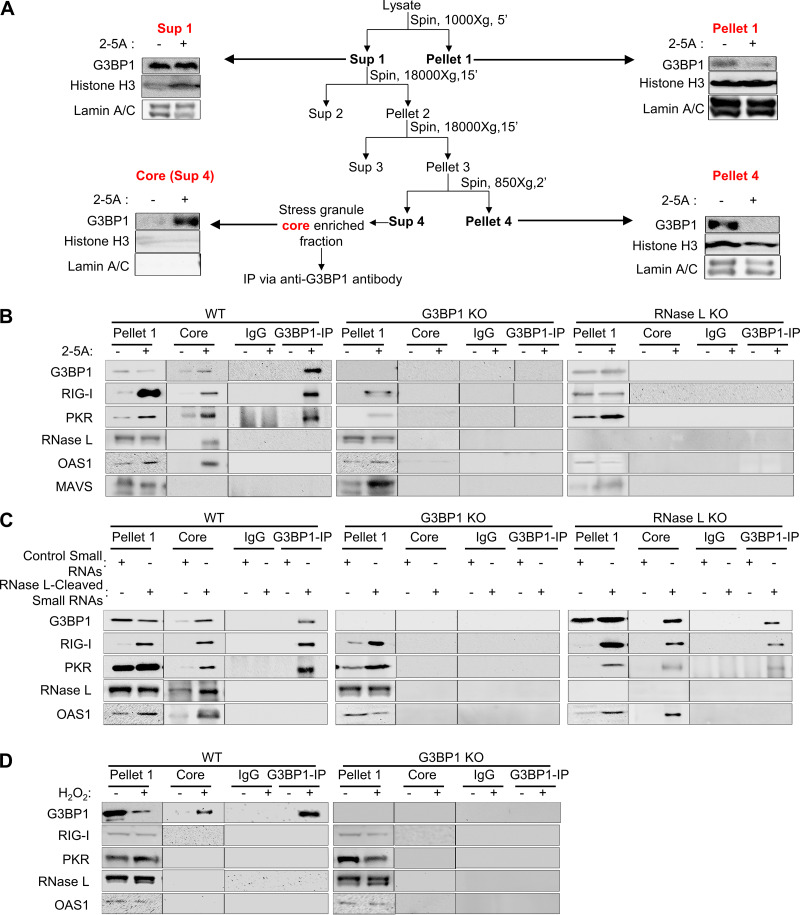
G3BP1 interacts with PKR and Rig-I, but not OAS and RNase L, in avSGs. (A) Schematic of avSG purification and analysis of fractions in immunoblots using the indicated antibodies. (B to D) HT1080 WT, G3BP1 KO, or RNase L KO cells were mock treated or treated with 10 μM 2-5A (B), treated with RNase L-cleaved small RNAs or control small RNAs (2 μg/ml) (C), or treated with 1 mM H_2_O_2_ or mock treated (D), and avSGs were isolated as described in Materials and Methods. The avSG core proteins were immunoprecipitated with G3BP1 antibody, and the immune complex was analyzed for the presence of PKR, Rig-I, OAS, RNase L, and MAVS (IPS-1) by immunoblot analysis. Pellet 1 and SG core (Sup 4) fractions were probed for expression of G3BP1 and PKR, Rig-I, OAS, RNase L, and MAVS (IPS-1). Nonspecific lanes were cropped to generate the images, and the boundaries are indicated. The data are representative of results from two experiments.

Studies have shown that avSGs provide a platform to coordinate viral sensing and IFN production by recruiting antiviral proteins and RNA ligands. We characterized avSGs formed during RNase L activation by adapting a method recently used to purify SG core from green fluorescent protein (GFP)-G3BP1-expressing cells with some changes (52). Expression of GFP-G3BP1 induced SGs independently of stimuli, so we used antibodies toward endogenous G3BP1 to immunoprecipitate and test the interaction of antiviral proteins from purified avSG core following 2-5A treatment in WT, G3BP1 KO, and RNase L KO cells (Fig. 3A and B). As PKR, Rig-I, OAS, and RNase L colocalized with G3BP1 upon 2-5A treatment, we examined if they purified with avSG core and tested physical interaction in the avSG core by coimmunoprecipitation. Cells were mock transfected or transfected with 2-5A, and avSG core was purified by multiple rounds of centrifugation as described in Materials and Methods. The cell pellet and core were analyzed for expression of PKR, Rig-I, OAS, and RNase L, and their interaction with G3BP1 was monitored in immune complexes by immunoblotting. Interaction of PKR and Rig-I with G3BP1 in the avSG core was seen only after 2-5A transfection; however, OAS and RNase L were present in the avSG core but did not interact with G3BP1. MAVS (IPS-1), a mitochondrial adaptor protein required for IFN production and RNase L-mediated IFN induction, was not present in the avSG core (Fig. 3B). As expected, cells lacking G3BP1 or RNase L did not assemble avSG core in response to 2-5A. These results are consistent with avSG colocalization and interaction of G3BP1 with PKR and Rig-I during IAVΔNS1 and NDV infection (35, 36). To further characterize avSG formation by RNase L-cleaved small RNAs, avSG core was purified from transfected cells and compared to control small RNAs (Fig. 3C). Consistent with avSG formation, RNase L-cleaved small RNAs promoted interaction of G3BP1 with PKR and Rig-I, as observed with 2-5A; however, both OAS and RNase L, while present in the core, do not interact with G3BP1. As expected, when RNase L-cleaved RNAs were introduced into RNase L KO cells, they induced formation of avSGs, and both PKR and Rig-I interacted with G3BP1 in avSG core, while cells lacking G3BP1 did not form avSGs (Fig. 3C). We compared these avSGs to the canonical SGs formed in response to oxidative stress by treating cells with H_2_O_2_ and analyzing the SG core for the presence of antiviral proteins. Consistent with our data shown in Fig. 2, WT cells formed SG cores, and none of the antiviral proteins were interacting or present in the SGs, while cells lacking G3BP1 did not form SG cores (Fig. 3D). These results allowed us to analyze the biochemical features of avSGs and revealed that, unlike the antiviral proteins OAS and RNase L, which are components of avSGs and do not interact with key proteins like G3BP1, PKR and Rig-I interact with G3BP1 and presumably form the scaffold and core of the SG. These results also raise the possibility of the existence of different types of complexes in the SG cores that coalesce to form a mature stress granule.

### avSG assembly by RNase L is required for IRF3-mediated interferon induction, but not for interferon signaling.

**FIG 4:**
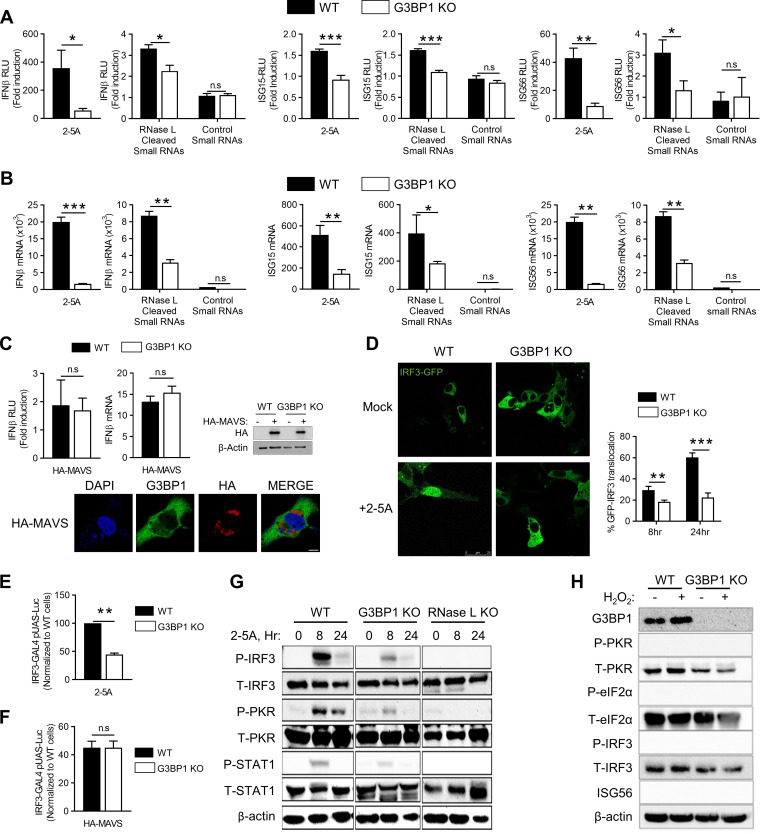
Antiviral SGs are required for IRF3-mediated IFN induction. (A) HT1080 WT and G3BP1 KO cells (1 × 10^5^) were transfected with IFN-β-luc, ISG15-luc, or ISG56-luc reporter constructs, along with β-galactosidase plasmids. After 24 h, the cells were treated with 10 μM 2-5A, 2 μg/ml of RNase L-cleaved small RNAs, or control small RNAs, and 8 h later, luciferase activity was measured and normalized to β-galactosidase levels. (B) HT1080 WT and G3BP1 KO cells were treated with 10 μM 2-5A, 2 μg/ml of RNase L-cleaved small RNAs, or control small RNAs, and 8 h later, IFN-β, ISG15, and ISG56 mRNA levels were measured by qRT-PCR and normalized to GAPDH mRNA levels. (C) WT and G3BP1 KO cells were transfected with empty vector or HA-MAVS(IPS-1), IFN-β-luc, and β-galactosidase plasmids, and after 24 h, promoter activity was normalized to β-galactosidase levels. The effects of HA-MAVS on IFN-β mRNA levels were determined by qRT-PCR, and HA-MAVS expression was confirmed in immunoblots. HA-MAVS-expressing cells were stained with G3BP1 to determine SG formation. (D) WT and G3BP1 KO cells were transfected with IRF3-GFP, and 24 h later, the cells were treated with 10 μM 2-5A or mock treated and imaged after 8 h. The percentages of cells with nuclear GFP-IRF3 were calculated in random fields from a minimum of 100 cells, and representative images are shown. (E and F) WT and G3BP1 KO cells were transfected with IRF3-GAL4 and UAS-luciferase plasmids and treated with 10 μM 2-5A (E) or HA-MAVS (F) for 8 h. The cells were lysed, and luciferase activity was measured. (G) WT, G3BP1 KO, or RNase L KO cells were transfected with 10 μM 2-5A for the indicated times, and P-IRF3, P-PKR, and P-STAT1 levels were determined in immunoblots and compared to unphosphorylated levels; β-actin was used to normalize loading. (H) WT and G3BP1 KO cells were transfected with 1 mM H_2_O_2_ for 3 h; levels of P-PKR, P-eIF2α, and P-IRF3 were compared to unphosphorylated levels, and induction of ISG56 was determined in immunoblots. The data represent means and standard errors (SE) for three independent experiments. *, *P* < 0.01; **, *P* < 0.001; ***, *P* < 0.0001; n.s, not significant.

Activation of RNase L by 2-5A produces dsRNA intermediates that signal through Rig-I and or MDA5 via the mitochondrial adaptor MAVS (IPS-1) by activating IRF3, which translocates to the nucleus to enhance IFN-β production (46). Various studies have shown that G3BP1 binds to Rig-I to regulate IFN-β production in response to viral RNA and synthetic dsRNA, poly(I·C) (39, 53, 54). To examine whether G3BP1 participates in RNase L-mediated IFN-β production, we monitored IFN-β promoter activation in WT and G3BP1 KO cells by directly activating RNase L with 2-5A or introducing RNase L-cleaved small RNAs compared to control small RNAs. In cells lacking G3BP1, IFN-β promoter activation was significantly reduced in response to both 2-5A and RNase L-cleaved small RNAs (Fig. 4A). Consequently, activation of promoters of ISGs, like ISG15 and ISG56/IFIT1, that are induced transcriptionally by IFN was also reduced. Consistent with the above-mentioned observations, mRNA levels of IFN-β, ISG15, and ISG56/IFIT1 were reduced in cells lacking G3BP1 in response to RNase L activation (Fig. 4B). Overexpression of MAVS (IPS-1) activates signaling pathways downstream of Rig-like receptors, resulting in phosphorylation and nuclear translocation of IRF3 to promote IFN-β production (18). In the absence of G3BP1, no difference in IFN-β promoter activation or IFN-β mRNA levels was observed in MAVS-overexpressing cells, suggesting avSGs function upstream of MAVS (IPS-1) (Fig. 4C). This is consistent with the absence of MAVS (IPS-1) in avSG core with RNase L activation (Fig. 3B). Furthermore, overexpression of MAVS (IPS-1) did not induce SG formation (Fig. 4C). To further investigate the requirement for avSGs in IRF3 activation, we monitored nuclear translocation of GFP-IRF3 in response to 2-5A for the indicated times in WT and G3BP1 KO cells by confocal microscopy. Loss of G3BP1 diminished GFP-IRF3 nuclear translocation 3-fold compared to WT cells (29% versus 60%) (Fig. 4D). We used a luciferase-based IRF3 transactivation assay to measure phosphorylation-dependent IRF3 activity. The assay uses the Gal4 DNA-binding domain and the IRF3 transactivation domain driving luciferase expression under the Gal4 promoter when IRF3 is phosphorylated (21). In G3BP1 KO cells, 2-5A induction of IRF3 transactivation was 44% that in WT cells expressing G3BP1 (Fig. 4E). As expected, overexpression of MAVS (IPS-1) resulted in similar levels of IRF3 transactivation independent of G3BP1 expression (Fig. 4F). The effect of G3BP1 on RNase L-mediated IFN-β production was apparent from reduced phosphorylation of PKR, IRF3, and STAT1 following 2-5A treatment in lysates of cells lacking G3BP1 or RNase L compared to strong activation in control WT cells (Fig. 4G). While activation of the dsRNA signaling pathway specifically promotes avSG assembly to induce IFN, H_2_O_2_ treatment forms SGs and does not activate PKR or IRF3 or produce IFN (Fig. 4H). Together, our data suggest that G3BP1 is essential for RNase L-mediated IFN induction by promoting the assembly of avSGs containing antiviral proteins and activating IRF3.

**FIG 5:**
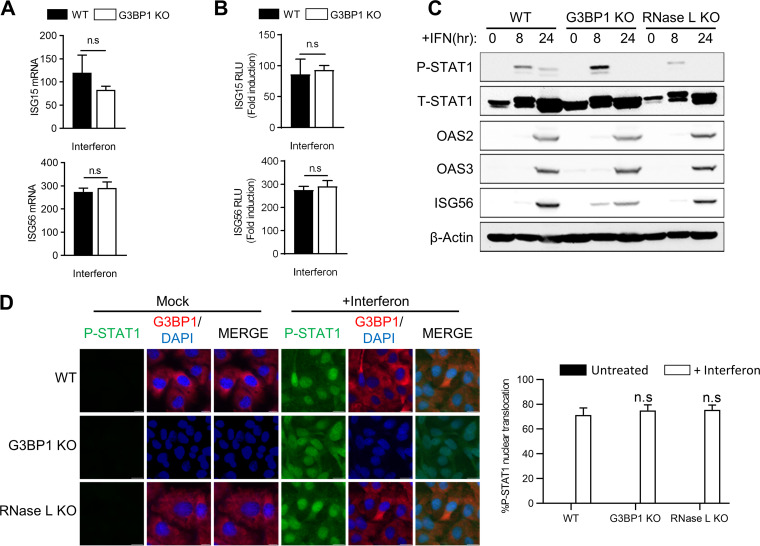
Effect of avSG formation on IFN signaling. (A and B) HT1080 WT and G3BP1 KO cells were treated with IFN-β (1,000 U/ml) for 24 h, and ISG15 and ISG56 mRNA levels were measured and normalized to those of GAPDH by qRT-PCR (A), or the cells were transfected with ISG15-luc or ISG56-luc reporter constructs, along with β-galactosidase plasmids, and 24 h later treated with IFN-β (1,000 U/ml), and luciferase activity was measured and normalized to β-galactosidase levels (B). (C) WT, G3BP1 KO, and RNase L KO cells were treated with IFN-β (1,000 U/ml) for the indicated times, and cell lysates were analyzed for phosphorylation of STAT1 and induction of OAS2, OAS3, and ISG56 in immunoblots. β-Actin was used to normalize loading. (D) WT, G3BP1 KO, and RNase L KO cells were treated with IFN-β (1,000 U/ml) for 16 h, and nuclear translocation of P-STAT1 was determined by immunofluorescence; nuclei were stained with DAPI. (Right) Quantification of P-STAT1 nuclear translocation from five random fields. The data represent means + SE for the results of three independent experiments. n.s, not significant.

IFN secreted by virus-infected cells binds to type I IFN receptor on the cell surface and activates the JAK-STAT signaling pathway, leading to transcriptional induction of several ISGs with roles in viral clearance mechanisms. Our results show the requirement for avSGs in producing IFN in response to RNase L activation; however, their role in IFN signaling is not clear. To examine this, we treated cells with type I IFN and monitored transcriptional induction of ISG15 and ISG56 using real-time PCR and promoter-driven luciferase reporter assays. In the absence of G3BP1, no significant differences in mRNA levels of both ISG15 and ISG56 or promoter-driven luciferase activity were observed upon IFN treatment (Fig. 5A and B). Exposure of WT, G3BP1 KO, or RNase L KO cells to type I IFN resulted in similar levels of phosphorylation of STAT1 accompanied by comparable levels of induction of ISGs, like OAS2, OAS3, and ISG56, in cell lysates on immunoblot analysis (Fig. 5C). Phosphorylated STAT1 translocates to the nucleus to induce transcription of genes regulated by IFN-stimulated response elements (ISRE) (3). No significant difference in phospho-STAT1 accumulation in the nucleus was observed with IFN treatment in cells lacking G3BP1 or RNase L compared to control WT cells (Fig. 5C and D). Taken together, these results indicate that avSG assembly, which requires G3BP1 protein, is required for IFN production in response to RNase L activation. However, following IFN production, G3BP1 is dispensable for activation of the JAK-STAT signaling pathway to transcriptionally induce ISGs.

### Induction of proinflammatory cytokines by RNase L is independent of antiviral stress granule assembly.

**FIG 6:**
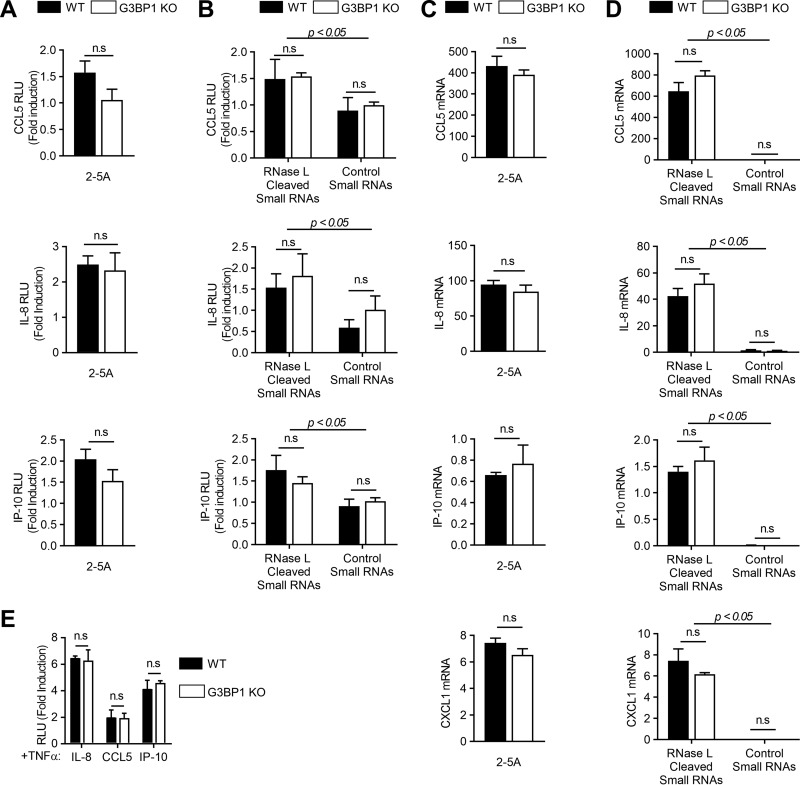
Induction of proinflammatory cytokines by RNase L is independent of avSG assembly. (A and B) WT and G3BP1 KO cells were transfected with CCL5-luc, IL-8-luc, or IP-10-luc and β-galactosidase plasmids and 24 h later treated with 2-5A (10 μM) (A) or RNase L-cleaved small RNAs or control small RNAs (B), and luciferase activity was normalized to β-galactosidase levels. (C and D) WT and G3BP1 KO cells were transfected with 2-5A (10 μM) (C) or RNase L-cleaved small RNAs or control small RNAs (D), and CCL5, IL-8, IP-10, and CXCL1 mRNA levels were measured by qRT-PCR and normalized to GAPDH mRNA levels. (E) WT and G3BP1 KO cells were transfected with CCL5-luc, IL-8-luc, or IP-10-luc and β-galactosidase plasmids and 24 h later treated with 100 ng/ml of TNF-α, and luciferase activity was normalized to β-galactosidase levels. *P* values for induction by RNase L-cleaved small RNAs compared to control small RNAs are shown. The data represent means + SE for three independent experiments. n.s, not significant.

Activation of RNase L or treatment with RNase L-cleaved RNAs induces inflammatory signaling pathways and proinflammatory cytokines (47, 55). We have demonstrated that avSGs are required for RNase L-mediated IFN production; however, the requirement for avSGs in inducing proinflammatory cytokines during RNase L activation is not known. To determine the effect of avSGs on cytokine induction during RNase L activation, we monitored CCL5 (RANTES), interleukin 8 (IL-8), or IP-10 promoter activation using luciferase reporter constructs in WT and G3BP1 KO cells by directly activating RNase L with 2-5A or introducing RNase L-cleaved small RNAs compared to control small RNAs. The 2-5A induction of CCL5 (RANTES), IL-8, or IP-10 promoter in G3BP1 KO cells was comparable to that in control WT cells (Fig. 6A). Consistent with the observation that RNase L-cleaved small RNAs promoted inflammasome signaling, we observed an increase in promoter activation in cells treated with these RNAs compared to control small RNAs. As with 2-5A treatment, depleting G3BP1 in cells did not affect induction of CCL5 (RANTES), IL-8, or IP-10 promoter by RNase L-cleaved small RNAs in promoter-driven luciferase assays (Fig. 6B). Similar increases in mRNA levels of CCL5 (RANTES), IL-8, or IP-10, as well as CXCL1, were observed in response to 2-5A and RNase L-cleaved RNAs in control WT cells, and depletion of G3BP1 did not affect mRNA levels as determined by real-time PCR analysis (Fig. 6C and D). To further analyze if tumor necrosis factor alpha (TNF-α)-induced cytokines are affected by SGs, we compared CCL5 (RANTES), IL-8, or IP-10 promoter induction in response to TNF-α in G3BP1 KO and control WT cells. No significant difference in promoter induction of TNF-α-induced cytokines was observed in cells lacking G3BP1 (Fig. 6E). These results show that while RNase L activation and the RNA cleavage products induce proinflammatory cytokines, unlike IFN-β production, avSGs induced by RNase L are not required for this effect, as cells lacking a key SG protein, G3BP1, induced comparable levels of the cytokines.

### Antiviral stress granule assembly restricts SeV replication.

**FIG 7:**
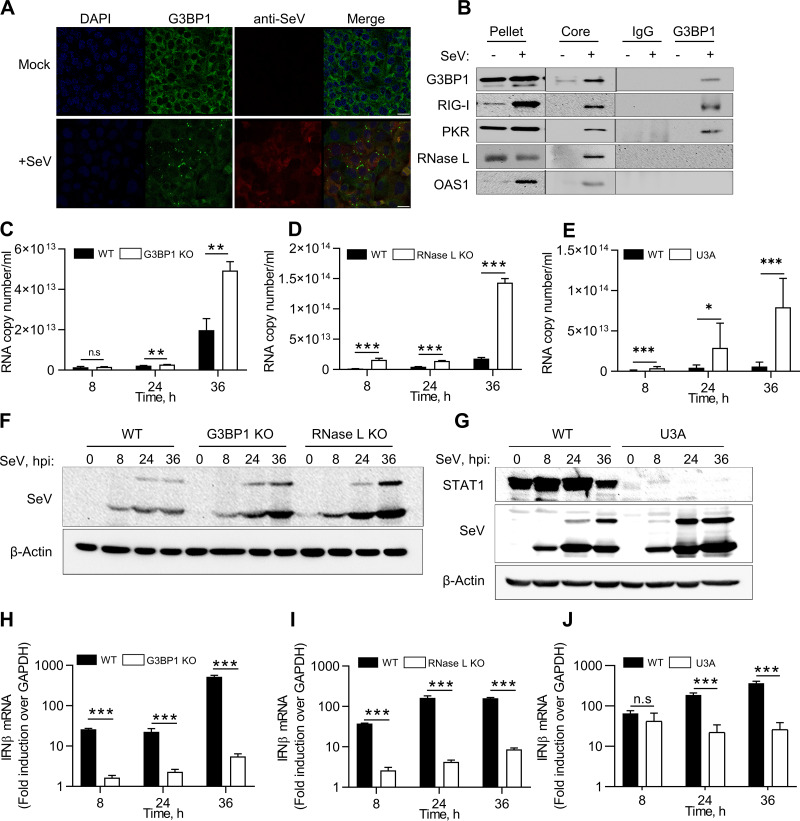
Antiviral roles of RNase L and G3BP1 during SeV infection. (A and B) WT cells were infected with SeV (40 HAU/ml) for 24 h, fixed, and stained with G3BP1 and antibody against SeV (A), and avSGs were purified as described in Materials and Methods (B). The avSG core proteins were immunoprecipitated with G3BP1 antibody, and the immune complex was analyzed for the presence of PKR, Rig-I, OAS, and RNase L by immunoblot analysis. Pellet and SG core fractions were probed for expression of G3BP1 and PKR, Rig-I, OAS, and RNase L. Nonspecific lanes were cropped to generate the images, and the boundaries are indicated. The data are representative of results from two experiments. (C to E) WT, G3BP1 KO, RNase L KO, or U3A (STAT1-defective) cells were infected with SeV (40 HAU/ml) for the indicated times, and viral titers were estimated by determining copy numbers of SeV genomic RNA strands in supernatants by qRT-PCR, (F to J) Expression of SeV antigens was detected using anti-Sendai virus antibody (F and G), and IFN-β mRNA levels were measured by qRT-PCR and normalized to GAPDH mRNA levels (H to J). The data represent means + SE for the results of three independent experiments. *, *P* < 0.01; **, *P* < 0.001; ***, *P* < 0.0001; n.s, not significant.

RNase L contributes to IFN-β production during Sendai virus (SeV) infection, and SeV is susceptible to RNase L antiviral effects (46). We tested the hypothesis that SeV infection induces avSG formation with antiviral roles in infected cells. Virus-infected cells were detected by immunostaining using anti-SeV antibodies for structural proteins 24 h postinfection, and SG formation was monitored by the appearance of G3BP1 puncta (Fig. 7A). To biochemically characterize the SGs formed during SeV infection as avSGs, we purified avSG core from infected cells, coimmunoprecipitated antiviral proteins that interacted with G3BP1, and compared them to those from uninfected cells. As with avSG formation in RNase L-activated cells, G3BP1 interacted with Rig-I and PKR in avSG core only during infection, and OAS and RNase L localized to avSGs but did not interact with G3BP1(Fig. 7B). We blocked the formation of avSGs to demonstrate their significance during SeV infection using cells lacking G3BP1, a protein critical for avSG assembly. To further understand the role of RNase L-induced avSGs, we used RNase L KO cells and compared SeV RNA copies produced during the time course of SeV infection up to 36 h. In G3BP1 KO cells, an increase in SeV RNA copies was observed at 24 h and further increased to 2.8-fold by 36 h compared to control WT cells (Fig. 7C). Consistent with previous studies, RNase L KO cells were more permissive to SeV replication, and numbers of SeV RNA copies were 5-fold higher at 24 h and a log unit higher 36 h postinfection (Fig. 7D). RNase L activity is regulated by IFN signaling, and dsRNA produced by RNase L amplifies IFN production via Rig-I/MAVS (IPS-1)/IRF3 pathways. To determine the role of IFN signaling in SeV infection, we infected U3A cells (STAT1-defective cells derived from HT1080 parental cells) with SeV and compared the numbers of SeV RNA copies produced up to 36 h after infection. Similar to other studies, lack of STAT1 protein in U3A cells resulted in greater replication of SeV (Fig. 7E) (56–59). The increase in viral titers in G3BP1, RNase L KO, and U3A cells was correlated with increased accumulation of SeV proteins during the time course of infection on immunoblots probed with anti-SeV antibodies (Fig. 7F and G). The increase in viral titers was correlated with a decrease in IFN-β produced during SeV infection in G3BP1 KO, RNase L KO, and U3A cells, demonstrating the importance of the antiviral role of RNase L, as well as avSG assembly, in SeV replication. Cells lacking STAT1 protein are defective in IFN signaling, demonstrating an important role in the SeV antiviral effect (Fig. 7H to J).

## DISCUSSION

RNase L is a regulated endoribonuclease that is activated in virus-infected cells by a unique ligand, 2-5A [p*x*5′A(2′p5′A)*n*; *x* = 1 to 3; *n* ≥ 2], to produce cleavage products that are predominantly double stranded with 5′ hydroxyl and 2′,3′-cyclic phosphate ends (60). RNase L-cleaved dsRNA activates signaling pathways by binding to diverse RNA-binding proteins to induce IFN-β, activate inflammasomes, induce autophagy, or promote a switch from autophagy to apoptosis (46–48, 61, 62). Previous studies showed that RNase L cleavage products amplify IFN-β production through Rig-I and or MDA5 via the MAVS (IPS-1) signaling pathway to sustain the antiviral response, but how the cells coordinate RNA sensing with the signaling response remains unclear (46). Our results show that RNase L activation induces avSGs containing a key stress granule protein, G3BP1, and the antiviral dsRNA-binding proteins Rig-I, PKR, and OAS, as well as RNase L, that are distinct from canonical SGs formed during oxidative stress (63). Our data using CRISPR/Cas9 knockout cells suggest these dsRNA products activate PKR, and subsequent phosphorylation of eIF2α induces avSGs, consistent with accumulation of dsRNA in SGs with G3BP1 in response to 2-5A. Biochemical analysis of avSGs using purified SGs revealed interaction of G3BP1 with Rig-I and PKR, which is consistent with avSGs being assembled in response to virus infection and dsRNA (35, 36, 39–41). OAS and RNase L, while present in avSG core, do not physically interact with G3BP1. Finally, we demonstrated the unique requirement for avSG assembly during RNase L activation for IRF3-mediated IFN-β induction, but not IFN signaling or induction of proinflammatory cytokines. Consequently, cells lacking avSG (G3BP1 KO) or RNase L signaling (RNase L KO) produced significantly less IFN during SeV infection and much higher viral titers due to a compromised antiviral response. We propose that during viral infection, RNase L contributes cleaved dsRNAs to induce avSGs that anchor antiviral dsRNA-binding proteins to provide a platform for efficient interaction of RNA ligands with pattern recognition receptors, like Rig-I, to enhance IFN-β production and antiviral response.

In our study, we transfected cells with 2-5A, a specific ligand to directly activate RNase L, and monitored the formation of unique SGs described as avSGs based on the recruitment of dsRNA-binding antiviral proteins like Rig-I, PKR, OAS, and RNase L. RNase L enzyme activity was required for avSG formation, as RNase L KO cells reconstituted with functional enzyme restored avSG formation while mutant RNase L that lacked nuclease activity did not. Similar to other reports, oxidative stress by H_2_O_2_ treatment induced the formation of canonical SGs that did not recruit antiviral proteins (63, 64). RNase L cleaves single-stranded viral and cellular RNAs after UU or UA residues, leaving 5′-hydroxyl and 2′,3′-cyclic phosphate termini on dsRNAs, which are required for IFN induction (46). PKR was activated by RNase L-cleaved RNAs by phosphorylating eIF2α, which in turn induced avSG formation. PKR KO cells lacked phospho-eIF2α in response to RNase L-cleaved RNAs, which was correlated with lack of avSG formation, while cells lacking Rig-I showed no effect. These results suggest that PKR is required for nucleation of avSGs by RNase L. Introducing RNA cleavage products into RNase L KO cells restored avSG formation similar to that of control WT cells, providing further evidence that RNase L-cleaved RNAs are inducers of avSGs by activating PKR. Removal of the 2′,3′-cyclic phosphate termini, which was required for IFN induction, decreased avSG formation, demonstrating correlation of avSG formation and IFN-inducing abilities. Also, dsRNA accumulated and colocalized with G3BP1 in SGs in cells treated with 2-5A. These results are consistent with avSGs formed in response to IAVΔNS1, NDV, encephalomyocarditis virus (EMCV), SINV, adenovirus, and hepatitis C virus (HCV) infection (26, 39, 65, 66). In other studies, formation of avSGs was also observed following transfection with synthetic dsRNA, poly(I·C), which has a broader effect by binding PKR, Rig-I, or OAS isoforms (67). Binding OAS results in 2-5A production from cellular ATP, which is the ligand for RNase L (10).

A recent report showed the formation of unique RNase L-dependent bodies (RLBs) distinct from SGs in cells treated with poly(I·C) (68). The RLB they identify is distinct from the avSGs we observed in that the RLBs were formed with poly(I·C) treatment in cells lacking G3BP1, were independent of SGs, and did not require PKR or phosphorylation of eIF2α, which were essential in our study for avSG formation. Also, the study did not explore if antiviral proteins localized with the RLBs they observed. These differences may be attributed to the use of poly(I·C), which can bind and activate other dsRNA-binding proteins, as described above. Furthermore, responses to poly(I·C) vary in a cell-type-dependent manner, depending on the levels of OAS isoforms, as well as the abundance of poly(I·C)-binding proteins in the cells (69). Recent reports show the role of RNase L in widespread mRNA degradation and translation repression of select basal mRNAs while antiviral mRNAs escaped decay and robustly translated (70, 71). These results suggest that RNA signaling and decay pathways activated by RNase L are complex and that the dynamics may vary based on specific activation of RNase L by 2-5A compared to indirect activation by poly(I·C), as well as cell type differences and the abundance of dsRNA-binding proteins, including OAS isoforms.

We characterized the biochemical nature of avSGs formed during RNase L activation using 2-5A, RNase L-cleaved RNAs, and SeV infection by adapting a recently published SG purification method and determined the interaction among proteins recruited to avSGs. Our studies avoided overexpression of G3BP1, which forms SGs independently of stimulus, by testing interaction with endogenous G3BP1 (52). Interestingly, only PKR and Rig-I interacted with G3BP1, while OAS and RNase L localized but did not interact. Recent studies have shown that mature stress granule cores recruit a shell that generates a liquid-liquid phase separation from the cytosol and forms a scaffold dominated by weak RNA-protein interactions (72). Further detailed analysis will be required to determine if OAS and RNase L are present in the shell that is dynamic while PKR and Rig-I interact with G3BP1 in the stable inner core. Several other RNA helicases, like DHX36, DDX3, and DDX6, and antiviral proteins, like ADAR1, ZAP, cGAS, and Trim25, localize in avSGs, suggesting cross talk between stress, RNA signaling, and antiviral pathways. Future studies will address the recruitment of these additional proteins and RNA ligands in avSGs and their relevance during a broad range of viral infections.

Cellular and viral RNA cleavage products generated by RNase L signal to the IFN-β gene through Rig-I/MDA5/MAVS (IPS-1) and IRF3 signaling pathways, and here, we showed involvement in inducing avSGs. We demonstrated a requirement for G3BP1, and thereby avSGs, in IRF3 activation and IFN production using G3BP1 KO cells. RNA cleavage products are primarily responsible for avSG formation and IFN-β induction, as their introduction into RNase L KO cells induced IFN-β while control RNAs had no effect. In response to viral infection, activated Rig-I interacts with MAVS (IPS-1) and is redistributed on mitochondria (17, 73). Accordingly, MAVS (IPS-1) did not localize in avSGs in our study, consistent with a similar lack of colocalization of Rig-I-containing avSGs with MAVS aggregates following IAVΔNS1 infection (35). Overexpression of MAVS (IPS-1) activated downstream signaling to activate IRF3, induced IFN-β independently of G3BP1 (Fig. 4F), and did not induce avSGs, indicating avSGs function upstream of MAVS signaling and IRF3 activation. Other stress-induced pathways, like oxidative stress, do not induce avSG formation or signaling events, as we have shown leading to IFN production, further demonstrating the distinct nature of avSGs and the signaling pathways activated. AvSG assembly is not required for IFN signaling, as IFN treatment induced ISG transcription in cells lacking G3BP1, like control WT cells. No difference in nuclear translocation of phospho-STAT1, which is required for type I IFN signaling, was observed in G3BP1 KO cells compared to control WT cells, consistent with the role of avSGs as a scaffold to recruit RNA sensors and PAMPs for signaling. RNase L also induces proinflammatory cytokines, and unexpectedly, cells lacking G3BP1 induced similar levels of proinflammatory cytokines in response to 2-5A and RNase L-cleaved RNAs. Induction of cytokines by TNF-α was also unaffected by lack of G3BP1. Consistent with our data, in prior studies, RNase L-cleaved RNAs stimulated NLRP3 complex formation and inflammasome activation to produce IL-1β by binding RNA helicase DHX33 and MAVS (IPS-1). Inflammasome activation was dependent on 2′,3′-cyclic phosphate termini on these RNAs and independent of both Rig-I and MDA5 but required MAVS (IPS-1) (47). Taken together, these results show a unique requirement for avSGs for IRF3-mediated IFN production distinct from proinflammatory cytokines. Both studies demonstrated bifurcation of RNA signaling pathways for proinflammatory cytokines from IFN production and an appearance of independence of Rig-like receptors but dependence on MAVS (IPS-1). In other studies, overexpression of GFP-G3BP1 in HeLa and U2OS cells induced SGs localizing innate immune proteins and regulated transcription through NF-κB and JNK, along with expression of cytokines (41). It is not clear if RNA ligand-induced avSG formation differs from overexpression of G3BP1 and if specific recruitment of PRRs results in specific activation of interferon versus other cytokines. Further detailed analysis of the biochemical features of the RNA ligands and the receptors will clarify how these pathways are specifically activated.

RNase L contributes to IFN-β production *in vivo* during SeV infection (46). Similar to RNase L KO cells, we observed reduced IFN-β production in cells lacking G3BP1 during SeV infection. Also, SeV infection induced the formation of SGs, which we characterized as avSGs, following purification and recruitment of PKR, Rig-I, OAS, and RNase L (Fig. 7A and B). Reduced levels of IFN-β production facilitated higher replication of SeV in both RNase L KO and G3BP1 KO cells, with loss of antiviral effect. Cells lacking STAT1 are defective in IFN signaling and transcriptional upregulation of ISGs with antiviral roles. We observed an increase in SeV replication and a decrease in IFN-β production consistent with studies that demonstrated inhibition of STAT1 signaling by direct binding of SeV C protein with STAT1 protein (58). While these observations suggest the importance of IFN signaling in SeV infection, further studies using diverse viruses would be needed to address how the RNase L-cleaved, RNA-cleaved products of the viral genomes participate in avSG formation and IFN production.

A recent study suggested that G3BP1 inhibits SeV and vesicular stomatitis virus (VSV) replication by suppressing RNF125-mediated ubiquitination of Rig-I, resulting in increased Rig-I expression and IFN production (54). These observations suggest that G3BP1 may regulate the host response to viral infections at multiple levels by regulating the activity of PRRs like Rig-I, as well as nucleating avSG formation. Prior studies showed that SeV infection produces highly structured dsRNA copy-back intermediates (defective viral genomes [DVGs]) with enhanced immunostimulatory activity (74). DVGs bind Rig-I and trigger expression of type I IFN and proinflammatory cytokines in infected cells (75). Another report identified unusual RNA species produced by various SeV strains, with IFN-inducing abilities that correlated with SG-like structures (76). While not explored in this study, we speculate that highly structured RNA motifs present in DVGs are released by RNase L activity, similar to RNA PAMPs produced from the 3′ region of the HCV genome during HCV infection to sustain IFN production (77).

RNase L has antiviral effects against a broad range of RNA and DNA viruses. We demonstrated a role for RNase L-cleaved RNAs in inducing stress granules to serve as an antiviral signaling hub by coordinating interaction of RNA ligands with PRRs to amplify IFN production and mount an effective antiviral response. It is likely that RNase L-cleaved RNAs are eventually turned over in P bodies harboring mRNA decay machinery to prevent sustained activation. Further studies will evaluate the role of RNase L-induced avSGs in the pathogenesis of viruses susceptible to RNase L antiviral effects and the broader impact on virus infection. Also, viruses antagonize the host response and SG assembly to promote replication in host cells. A critical balance of host stress response pathways and viral manipulation of these pathways eventually dictates the outcome of viral infections.

## MATERIALS AND METHODS

### Chemicals, reagents, and antibodies.

Chemicals, unless otherwise indicated, were from Sigma-Aldrich (St. Louis, MO, USA). Antibodies against G3BP1 (SC-81940), OAS1 (SC-98424), OAS2 (SC-374238), RNase L (SC-22870), PKR (SC-707), and RIG-I (SC-48931) were from Santa Cruz Biotechnology. The G3BP1 (A302-033A) used for immunoprecipitation was from Bethyl Laboratories. Sendai virus (MBL-PD029) was from MBL. FLAG (14793), phospho-eIF2α (3398), eIF2α (5324), histone H3 (9715), lamin A/C (4777), phospho-IRF3 (4947), IRF3 (4302), phospho-STAT1 (9167), STAT1 (9172), ISG56 (14769), and β-actin (3700) were from Cell Signaling Technology. Recombinant human TNF-α (PHC3011) and antibody against OAS3 (PA5-31090) were from Thermo Fisher Scientific. RIG-I (ALX-804-849-C100) and MAVS (ALX-210-929-C100) were from Enzo Life Sciences. Phospho-PKR (AB81303) and total PKR (1511-1) were from Abcam. Monoclonal antibody to human RNase L was kindly provided by Robert Silverman (Cleveland Clinic); dsRNA (J2) was from English & Scientific Consulting. Anti-mouse IgG and anti-rabbit IgG horseradish peroxidase (HRP)-linked secondary antibodies were from Cell Signaling Technology, and enhanced chemiluminescence (ECL) reagents were from Boston Bioproducts and GE Healthcare. IFN-β was from Biogen Idec. Hydrogen peroxide (H325-100) and puromycin (BP2956100) were purchased from Fisher Scientific.

### 2-5A synthesis using recombinant OAS1.

2-5A was prepared enzymatically from ATP and recombinant porcine 2-5A synthetase (a generous gift from Rune Hartmann, University of Aarhus, Aarhus, Denmark) activated with poly(I·C) conjugated to agarose beads as described previously (47, 78, 79). Poly(I·C)-agarose was incubated with 0.4 mg/ml of purified OAS1 at 25°C in buffer containing 10 mM HEPES, pH 7.5, 1.5 mM magnesium acetate, 20% glycerol, 50 mM KCl, 7 mM β-mercaptoethanol for 1 h. After 1 h, the OAS1-immobilized beads were washed three times in the same buffer and incubated in buffer containing 10 mM ATP, 1.5 mM magnesium acetate, 10% glycerol, 50 mM KCl, and 7 mM β-mercaptoethanol for 20 h at 37°C with gentle shaking at 150 rpm on a platform shaker. The crude 2-5A preparation was clarified by centrifugation at 3,000 × *g* for 15 min. The 2-5A oligomers were further separated from OAS1 and any contaminating poly(I·C) using Centriprep (Millipore) with a 3-kDa cutoff. We confirmed lack of poly(I·C) in our 2-5A preparations by using a parallel small-scale reaction mixture that was spiked with a known amount of poly(I·C) and passed through Centriprep at 3 kDa. The 2-5A preparation obtained was a mixture of ppp(A2′p)*_n_*A in the following proportions: *n* = 1, 16%; *n* = 2, 37%; *n* = 3, 26%; and *n* = 4, 9%. It was applied to cells by complexing with lipofectamine 2000 reagent according to the manufacturer’s recommendations.

### Cell culture and transfections.

The human fibrosarcoma cell line HT1080 (a gift from Ganes Sen, Cleveland Clinic, Cleveland, OH, USA) and U3A (STAT1 defective; derived from parental 2fTGH cells, which were derived from HT1080 cells; a gift from George Stark, Cleveland Clinic), G3BP1 KO, RNase L KO, PKR KO, Rig-I KO, and PKR/Rig-I KO cells were cultured in Dulbecco’s modified minimal essential medium with 10% fetal bovine serum,100 μg/ml penicillin/streptomycin, 2 mM l-glutamine, and nonessential amino acids. The cells were maintained in 95% air, 5% CO_2_ at 37°C. Transfection of 2-5A (10 μM) was performed using Lipofectamine 2000 (Invitrogen, Carlsbad, CA, USA) according to the manufacturer’s protocol. RNase L-cleaved small RNAs and control small RNAs were prepared as previously described (46, 80) and transfected (2 μg/ml) using Polyjet reagent (SignaGen Laboratories) according to the manufacturer’s protocol. H_2_O_2_ (1 μM) was added to cell culture media for 3 h to induce oxidative stress.

### Generation of cells with *PKR*, *Rig-I*, *RNase L*, and *G3BP1* knockout using the CRISPR/Cas9 system.

Knockout cells were generated using the CRISPR/Cas9 system (80, 81). Small guide RNAs (sgRNAs) (Table 1) were designed using GPP sgRNA Designer (https://portals.broadinstitute.org/gpp/public/analysis-tools/sgrna-design) (8). The guide RNA sequences were synthesized as DNA oligonucleotides and annealed, phosphorylated, and ligated into the vector pSpCas9(BB)-2A-Puro (PX459; Addgene plasmid 62988) V2.0 (a gift from Feng Zhang), which was prepared by digestion with BsmBI. HT1080 cells (3 × 10^5^ cells/well of a 6-well plate) were transfected with 2 μg of the resulting plasmids and selected in 1 μg/ml puromycin. Clones were obtained by limiting dilution, and gene knockout colonies were validated by immunoblotting and sequencing (Table 2).

**TABLE 1:** sgRNA sequences for RNase L, G3BP1, PKR, and Rig-I knockout using the CRISPR/Cas9 system

Gene	Orientation	Sequence
RNase L	Forward	5′-CACCGCAATCGCTGCGAGGATAAA-3′
	Reverse	5′-AAACTTTATCCTCGCAGCGATTGC-3′
G3BP1	Forward	5′-CACCGAATTCCCGCCCGACCAGCAG-3′
	Reverse	5′-AAACCTGCTGGTCGGGCGGGAATTC-3′
EIF2AK2 (PKR)	Forward	5′-CACCGCAGGACCTCCACATGATAGG-3′
	Reverse	5′-AAACCCTATCATGTGGAGGTCCTGC-3′
DDX58 (RIG-I)	Forward	5′-CACCGGGATTATATCCGGAAGACCC-3′
	Reverse	5′-AAACGGGTCTTCCGGATATAATCCC-3′

**TABLE 2:** Schematic presentation of the coding regions of RNase L, G3BP1, RIG-I, PKR, and RIG-I/PKR dKO that are targeted by CRISPR-Cas9

Gene	Sequence type^*a*^	Sequence^*b*^
RNase L	REF	ATGGGGCCACGCCT**TTTATCCTCGCAGCGATTGCGGGG** AGCGTGAAGCTGCTGAAACTTTTCCT
	MUT	ATGGGGCCACGCCTTTTATCCTC ----------------------- GCTGCTGAAACTTTTCCT
G3BP1	REF	GGTTGAATTGACCAAAGCAATGGTGATGGAGAAGCCTAGTCCC**CTGCTGGTCGGGCGGGAAT** TTGTGA
	MUT	GGTTGAATT ------------------------------------------------- GAATTTGTGA
RIG-I	REF	TTCCAGGATTATATCCGGAAG-* ACCCTGG ACCCTACCTACA
	MUT	TTCCAGGATTATATCCGGAAGAACCCTGGACCCTACCTACA
PKR	REF	CTAATTCAGGACCTCCACATGA-* TAGGAGG TAGGTTGC
	MUT	CTAATTCAGGACCTCCACATGATTAGGAGGTAGGTTGC
RIG-I/PKR dKO^*c*^	REF	TTCCAGGATTATATCCGGAAG-* ACCCTGG ACCCTACCTACA
	MUT	TTCCAGGATTATATCCGGAAGAACCCTGGACCCTACCTACA
	REF	CTAATTCAGGACCTCCACATGA-* TAGGAGG TAGGTTGC
	MUT	CTAATTCAGGACCTCCACATGATTAGGAGGTAGGTTGC

a REF, reference sequence; MUT, mutated sequence.

b The reference sequence and the mutated sequence for each gene are shown, as confirmed by sequencing. The boldface letters denote the protospacer (sgRNA-binding site); the underlined letters indicate the PAM (protospacer-adjacent motif).

c The top two sequences are RIG-I; the bottom two sequences are PKR.

### Plasmids.

Plasmids Flag-RNase L, Flag-RNase L R667A (Robert Silverman, Cleveland Clinic), GFP-IRF3 (Travis Taylor, University of Toledo), HA-IPS-1(MAVS) (Invivogen), IFN-β-luc (Michael Gale, University of Washington), ISG56-luc (Ganes Sen, Cleveland Clinic), ISG15-luc (Bret Hassel, University of Maryland), IP10-luc and IL-8-luc (George Stark, Cleveland Clinic), and CCL5-luc and IRF3-Gal/UAS-luc (Katherine Fitzgerald, University of Massachusetts) were transfected using Polyjet reagent according to the manufacturer’s instructions.

### Western blot analysis.

Cells were lysed in NP-40 lysis buffer containing 0.5% NP-40, 90 mM KCl, 5 mM magnesium acetate, 20 mM Tris, pH 7.5, 5 mM β-mercaptoethanol, 0.1 M phenylmethylsulfonyl fluoride (PMSF), 0.2 mM sodium orthovanadate, 50 mM NaF, 10 mM glycerophosphate, and protease inhibitor (Roche Diagnostics). The lysates were clarified by centrifugation at 10,000 × *g* (4°C for 20 min). Proteins (15 to 100 μg per lane) were separated in polyacrylamide gels containing SDS, transferred to nitrocellulose membranes (Bio-Rad), and probed with different primary antibodies according to the manufacturer’s protocols. The membranes were incubated with goat anti-mouse or goat anti-rabbit antibody tagged with horseradish peroxidase (Cell Signaling), and immunoreactive bands were detected by enhanced chemiluminescence (GE Healthcare and Boston Bioproducts). Images were processed using Adobe Photoshop CS4 (Adobe, San Jose, CA, USA). In some instances, nonspecific lanes were cropped to generate the images, and the boundaries are indicated in representative figures.

### Immunofluorescence analysis.

Cells were cultured on glass coverslips, and after treatment, the cells were fixed with 4% paraformaldehyde (Boston Bioproducts) for 15 min and permeabilized with 0.1% Triton X-100 in phosphate-buffered saline (PBS) for 15 min. The cells were then blocked with 3% bovine serum albumin (BSA) for 1 h at room temperature and incubated overnight at 4°C with the indicated antibodies. Alexa488- or Alexa647-conjugated anti-immunoglobulin antibody (Molecular Probes) was used as a secondary antibody. Cell nuclei were stained with Vectashield, with DAPI (4′,6-diamidino-2-phenylindole) to stain the nucleus (Vector Laboratories). Fluorescence and confocal microscopy assessments were performed with a Leica CS SP5 multiphoton laser scanning confocal microscope (Leica Microsystems). All subsequent analysis and processing of images were performed using LAS AF software (Leica Microsystems). Cells containing avSGs (*n* > 5) that were above 0.6 μm in diameter were considered for analysis. The percentages of avSG-containing cells were calculated in at least five random fields from a minimum of 100 cells per treatment. Colocalization of proteins in stress granules was assessed by line scan analysis using Image J as described previously (82). A line was drawn across the stress granules, and the intensities were measured using a plot profile. The arbitrary intensity was plotted according to an arbitrary distance for each channel.

### RNA isolation, rRNA cleavage assay, and quantitative real-time PCR.

Total RNA was isolated from cells using TRIzol reagent (Invitrogen) according to the manufacturer’s instructions and resolved on RNA chips using a Bioanalyzer 2100 (Agilent Technologies) as described previously (62). Reverse transcription and cDNA synthesis were performed using random decamers and a Retroscript cDNA synthesis kit (Life Technologies; Thermo Fisher Scientific). Gene expression was determined by quantitative reverse transcription PCR (qRT-PCR) using SYBR green PCR master mix (Bio-Rad Laboratories Inc., Hercules, CA, USA) with gene-specific primers (Table 3) and normalized to GAPDH (glyceraldehyde-3-phosphate dehydrogenase) expression.

**TABLE 3:** Primer sequences for RT-PCR

Primer	Orientation	Sequence
IFNB1	Forward	5′-GGAGGACGCCGCATTGAC-3′
	Reverse	5′-TGATAGACATTAGCCAGGAGGTTC-3′
ISG15	Forward	5′-TGCAGAACTGCATCTCCATC-3′
	Reverse	5′-TTCATGAGGCCGTATTCCTC-3′
IFIT1 (ISG56)	Forward	5′-TACAGCAACCATGAGTACAA-3′
	Reverse	5′-TCAGGTGTTTCACATAGGC-3′
CCL5	Forward	5′-CCAGCAGTCGTCTTTGTCAC-3′
	Reverse	5′-CTCTGGGTTGGCACACACTT-3′
CXCL8 (IL-8)	Forward	5′-AAGAGAGCTCTGTCTGGACC-3′
	Reverse	5′-GATATTCTCTTGGCCCTTGG-3′
CXCL10 (IP-10)	Forward	5′-TTCCTGCAAGCCAATTTTGTC-3′
	Reverse	5′-TCTTCTCACCCTTCTTTTTCATTGT-3′
CXLC1	Forward	5′-GCGCCCAAACCGAAGTCATA-3′
	Reverse	5′-ATGGGGGATGCAGGATTGAG-3′
SeV	Forward	5′-GACGCGAGTTATGTGTTTGC-3′
	Reverse	5′-TTCCACGCTCTCTTGGATCT-3′
GAPDH	Forward	5′-GCAAATTCCATGGCACCGT-3′
	Reverse	5′-TCGCCCCACTTGATTTTGG-3′

### Isolation of RNase L-cleaved small RNAs and control small RNAs.

RNase L-cleaved small RNAs and control small RNAs were prepared as previously described with modifications as follows (46, 47, 77). Total cellular RNA was isolated from HT1080 cells transfected with 10 μM 2-5A (the source of RNase L-cleaved small RNAs) or mock-transfected cells (control small RNAs) using TRIzol reagent after 6 h. RNase L activation, as determined by rRNA cleavage, was determined on RNA chips prior to isolation of RNase L-cleaved small RNAs. Small-RNA cleavage products (<200 nucleotides [nt]) were purified using a solid-phase fractionation method (mirVana miRNA isolation kit; Ambion) as described previously (46, 47, 77). Briefly, total RNA isolated from cells (treated with 2-5A or mock treated) was mixed with 1.25 volumes of 100% ethanol, and RNA bound to the column filter was washed with buffers according to the manufacturer’s recommendation. The bound RNA, which represents RNAs of <200 nt, was eluted in nuclease-free water. The enrichment of small RNAs was verified on RNA chips. The lack of 2-5A in the small RNAs purified was confirmed by fluorescence resonance energy transfer (FRET) assay (83) using reagents provided by Robert Silverman (Cleveland Clinic) and by monitoring degradation of total RNA incubated with purified RNase L and 2-5A as a positive control on RNA chips. Small RNAs lacking the 2′-3′-phosphoryl groups were generated by incubating with 10 U calf intestinal phosphatase (NEB) for 1 h at 37°C, followed by 10 min at 75°C, and compared to control reactions, as previously described (46, 77). The small RNAs were again purified using a solid-phase fractionation method (mirVana miRNA isolation kit; Ambion), analyzed on RNA chips, and applied to cells by complexing with Polyjet reagent (SignaGen Laboratories) according to the manufacturer’s protocol.

### Luciferase assay.

Cells (1 × 10^5^) were seeded in a 12-well plate and transfected with the indicated plasmids, along with pCH110 β-galactosidase-expressing plasmid to normalize transfection efficiency. Cells were harvested at the indicated time points in luciferase lysis buffer, and luciferase activity was determined using luciferase reagents (Goldbio; USA) and normalized to β-galactosidase levels (84).

### Stress granule isolation and immunoprecipitation.

Stress granules were isolated as described previously (52). Briefly, cells were grown on six 10-cm dishes, and after stress, the cells were pelleted at 1,500 × *g* for 3 min. Upon removal of the medium, the pellets were immediately flash frozen in liquid N_2_ and stored at −80°C until isolation of the stress granule core was performed. The cell pellet was thawed on ice for 5 min, resuspended in 1 ml of SG lysis buffer (50 mM Tris-HCl [pH 7.4], 100 mM potassium acetate, 2 mM magnesium acetate, 0.5 mM dithiothreitol, 50 μg/ml heparin, 0.5% NP-40, EDTA-free protease inhibitor, 1 U/μl of RNasin plus RNase inhibitor [Promega]), and passed through a 25-gauge 5/8 needle attached to a 1-ml syringe 10 times. After lysis, the lysates were spun for 5 min at 1,000 × *g* at 4°C to remove cell debris. The supernatant was spun at 18,000 × *g* for 20 min at 4°C to pellet the SG core. The resulting supernatant was discarded, and the pellet was resuspended in 1 ml of SG lysis buffer and spun at 18,000 × *g* for 20 min at 4°C. The resulting pellet was resuspended in 300 μl of SG lysis buffer and spun at 850 × *g* for 2 min at 4°C. The supernatant, which represented the SG core-enriched fraction, was transferred to a new tube. Equal amounts of SG core were subjected to immunoprecipitation using anti-G3BP1 antibody (1 μg), isotype-specific control antibody, and protein A-Sepharose beads (Sigma-Aldrich). Samples were incubated at 4°C overnight on a rotator, and immune complexes were recovered by centrifugation and five washes in buffer. Samples were boiled in SDS sample buffer and analyzed by protein gel electrophoresis and immunoblotting using the indicated antibodies.

### Viral growth kinetics.

Cells (5 × 10^5^) were plated in a 6-well plate, and the next day, the cells were infected with Sendai virus (strain Cantell; Charles Rivers Laboratories) at 40 hemagglutinating units (HAU)/ml in medium without serum. After 1 h, the medium was replaced with complete medium, and cells were harvested at the indicated time points. Expression of viral antigen was determined on Western blots using anti-Sendai virus antibody. Total RNA was isolated from infected cells using TRIzol reagent (Invitrogen) or a QIAmp viral RNA kit (Qiagen), and qRT-PCR was performed to quantify the viral RNA copy number as described previously (62).

### Statistical analysis.

All values are presented as means and standard errors of the mean (SEM) from at least three independent experiments or are representative of three independent experiments performed in triplicate and are shown as means and standard deviations (SD). Student’s *t* tests were used for determining statistical significance between groups, using Prism8 (GraphPad) software, and *P* values of <0.05 were considered significant.

## References

[1] Kawai T, Akira S 2006 Innate immune recognition of viral infection. Nat Immunol 7:131–137. doi:10.1038/ni1303. 16424890

[2] Kawai T, Akira S 2011 Regulation of innate immune signalling pathways by the tripartite motif (TRIM) family proteins. EMBO Mol Med 3:513–527. doi:10.1002/emmm.201100160. 21826793 PMC3377094

[3] Borden EC, Sen GC, Uze G, Silverman RH, Ransohoff RM, Foster GR, Stark GR 2007 Interferons at age 50: past, current and future impact on biomedicine. Nat Rev Drug Discov 6:975–990. doi:10.1038/nrd2422. 18049472 PMC7097588

[4] Koyama S, Ishii KJ, Coban C, Akira S 2008 Innate immune response to viral infection. Cytokine 43:336–341. doi:10.1016/j.cyto.2008.07.009. 18694646

[5] Medzhitov R 2007 TLR-mediated innate immune recognition. Semin Immunol 19:1–2. doi:10.1016/j.smim.2007.02.001. 22228983 PMC3252746

[6] Loo YM, Gale M Jr 2011 Immune signaling by RIG-I-like receptors. Immunity 34:680–692. doi:10.1016/j.immuni.2011.05.003. 21616437 PMC3177755

[7] Zhang Z, Kim T, Bao M, Facchinetti V, Jung SY, Ghaffari AA, Qin J, Cheng G, Liu YJ 2011 DDX1, DDX21, and DHX36 helicases form a complex with the adaptor molecule TRIF to sense dsRNA in dendritic cells. Immunity 34:866–878. doi:10.1016/j.immuni.2011.03.027. 21703541 PMC3652560

[8] Yim HC, Williams BR 2014 Protein kinase R and the inflammasome. J Interferon Cytokine Res 34:447–454. doi:10.1089/jir.2014.0008. 24905201

[9] Balachandran S, Barber GN 2007 PKR in innate immunity, cancer, and viral oncolysis. Methods Mol Biol 383:277–301. doi:10.1007/978-1-59745-335-6_18. 18217692

[10] Silverman RH 2007 A scientific journey through the 2-5A/RNase L system. Cytokine Growth Factor Rev 18:381–388. doi:10.1016/j.cytogfr.2007.06.012. 17681844 PMC2075094

[11] Silverman RH 2007 Viral encounters with 2′,5′-oligoadenylate synthetase and RNase L during the interferon antiviral response. J Virol 81:12720–12729. doi:10.1128/JVI.01471-07. 17804500 PMC2169107

[12] Dempsey A, Bowie AG 2015 Innate immune recognition of DNA: a recent history. Virology 479-480:146–152. doi:10.1016/j.virol.2015.03.013. 25816762 PMC4424081

[13] Hopfner KP 2014 RIG-I holds the CARDs in a game of self versus nonself. Mol Cell 55:505–507. doi:10.1016/j.molcel.2014.08.009. 25148359

[14] Yoneyama M, Fujita T 2009 RNA recognition and signal transduction by RIG-I-like receptors. Immunol Rev 227:54–65. doi:10.1111/j.1600-065X.2008.00727.x. 19120475

[15] Jiang X, Kinch LN, Brautigam CA, Chen X, Du F, Grishin NV, Chen ZJ 2012 Ubiquitin-induced oligomerization of the RNA sensors RIG-I and MDA5 activates antiviral innate immune response. Immunity 36:959–973. doi:10.1016/j.immuni.2012.03.022. 22705106 PMC3412146

[16] Arimoto K, Takahashi H, Hishiki T, Konishi H, Fujita T, Shimotohno K 2007 Negative regulation of the RIG-I signaling by the ubiquitin ligase RNF125. Proc Natl Acad Sci U S A 104:7500–7505. doi:10.1073/pnas.0611551104. 17460044 PMC1863485

[17] Kawai T, Takahashi K, Sato S, Coban C, Kumar H, Kato H, Ishii KJ, Takeuchi O, Akira S 2005 IPS-1, an adaptor triggering RIG-I- and Mda5-mediated type I interferon induction. Nat Immunol 6:981–988. doi:10.1038/ni1243. 16127453

[18] Seth RB, Sun L, Ea CK, Chen ZJ 2005 Identification and characterization of MAVS, a mitochondrial antiviral signaling protein that activates NF-kappaB and IRF 3. Cell 122:669–682. doi:10.1016/j.cell.2005.08.012. 16125763

[19] Xu LG, Wang YY, Han KJ, Li LY, Zhai Z, Shu HB 2005 VISA is an adapter protein required for virus-triggered IFN-beta signaling. Mol Cell 19:727–740. doi:10.1016/j.molcel.2005.08.014. 16153868

[20] Meylan E, Curran J, Hofmann K, Moradpour D, Binder M, Bartenschlager R, Tschopp J 2005 Cardif is an adaptor protein in the RIG-I antiviral pathway and is targeted by hepatitis C virus. Nature 437:1167–1172. doi:10.1038/nature04193. 16177806

[21] Fitzgerald KA, McWhirter SM, Faia KL, Rowe DC, Latz E, Golenbock DT, Coyle AJ, Liao SM, Maniatis T 2003 IKKepsilon and TBK1 are essential components of the IRF3 signaling pathway. Nat Immunol 4:491–496. doi:10.1038/ni921. 12692549

[22] McWhirter SM, Fitzgerald KA, Rosains J, Rowe DC, Golenbock DT, Maniatis T 2004 IFN-regulatory factor 3-dependent gene expression is defective in Tbk1-deficient mouse embryonic fibroblasts. Proc Natl Acad Sci U S A 101:233–238. doi:10.1073/pnas.2237236100. 14679297 PMC314168

[23] Balachandran S, Roberts PC, Brown LE, Truong H, Pattnaik AK, Archer DR, Barber GN 2000 Essential role for the dsRNA-dependent protein kinase PKR in innate immunity to viral infection. Immunity 13:129–141. doi:10.1016/s1074-7613(00)00014-5. 10933401

[24] Taylor SS, Haste NM, Ghosh G 2005 PKR and eIF2alpha: integration of kinase dimerization, activation, and substrate docking. Cell 122:823–825. doi:10.1016/j.cell.2005.09.007. 16179248

[25] Anderson P, Kedersha N 2008 Stress granules: the Tao of RNA triage. Trends Biochem Sci 33:141–150. doi:10.1016/j.tibs.2007.12.003. 18291657

[26] Onomoto K, Yoneyama M, Fung G, Kato H, Fujita T 2014 Antiviral innate immunity and stress granule responses. Trends Immunol 35:420–428. doi:10.1016/j.it.2014.07.006. 25153707 PMC7185371

[27] Youn JY, Dunham WH, Hong SJ, Knight JDR, Bashkurov M, Chen GI, Bagci H, Rathod B, MacLeod G, Eng SWM, Angers S, Morris Q, Fabian M, Cote JF, Gingras AC 2018 High-density proximity mapping reveals the subcellular organization of mRNA-associated granules and bodies. Mol Cell 69:517–532 e11. doi:10.1016/j.molcel.2017.12.020. 29395067

[28] Nover L, Scharf KD, Neumann D 1989 Cytoplasmic heat shock granules are formed from precursor particles and are associated with a specific set of mRNAs. Mol Cell Biol 9:1298–1308. doi:10.1128/mcb.9.3.1298. 2725500 PMC362722

[29] Piotrowska J, Hansen SJ, Park N, Jamka K, Sarnow P, Gustin KE 2010 Stable formation of compositionally unique stress granules in virus-infected cells. J Virol 84:3654–3665. doi:10.1128/JVI.01320-09. 20106928 PMC2838110

[30] Williams BR 2001 Signal integration via PKR. Sci STKE 2001:re2. doi:10.1126/stke.2001.89.re2. 11752661

[31] Buchan JR, Parker R 2009 Eukaryotic stress granules: the ins and outs of translation. Mol Cell 36:932–941. doi:10.1016/j.molcel.2009.11.020. 20064460 PMC2813218

[32] Jain S, Wheeler JR, Walters RW, Agrawal A, Barsic A, Parker R 2016 ATPase-modulated stress granules contain a diverse proteome and substructure. Cell 164:487–498. doi:10.1016/j.cell.2015.12.038. 26777405 PMC4733397

[33] Wheeler JR, Matheny T, Jain S, Abrisch R, Parker R 2016 Distinct stages in stress granule assembly and disassembly. Elife 5:e18413. doi:10.7554/eLife.18413. 27602576 PMC5014549

[34] Hu S, Sun H, Yin L, Li J, Mei S, Xu F, Wu C, Liu X, Zhao F, Zhang D, Huang Y, Ren L, Cen S, Wang J, Liang C, Guo F 2019 PKR-dependent cytosolic cGAS foci are necessary for intracellular DNA sensing. Sci Signal 12:eaav7934. doi:10.1126/scisignal.aav7934. 31772125

[35] Onomoto K, Jogi M, Yoo JS, Narita R, Morimoto S, Takemura A, Sambhara S, Kawaguchi A, Osari S, Nagata K, Matsumiya T, Namiki H, Yoneyama M, Fujita T 2012 Critical role of an antiviral stress granule containing RIG-I and PKR in viral detection and innate immunity. PLoS One 7:e43031. doi:10.1371/journal.pone.0043031. 22912779 PMC3418241

[36] Yoo JS, Takahasi K, Ng CS, Ouda R, Onomoto K, Yoneyama M, Lai JC, Lattmann S, Nagamine Y, Matsui T, Iwabuchi K, Kato H, Fujita T 2014 DHX36 enhances RIG-I signaling by facilitating PKR-mediated antiviral stress granule formation. PLoS Pathog 10:e1004012. doi:10.1371/journal.ppat.1004012. 24651521 PMC3961341

[37] Rozelle DK, Filone CM, Kedersha N, Connor JH 2014 Activation of stress response pathways promotes formation of antiviral granules and restricts virus replication. Mol Cell Biol 34:2003–2016. doi:10.1128/MCB.01630-13. 24662051 PMC4019067

[38] Thulasi Raman SN, Liu G, Pyo HM, Cui YC, Xu F, Ayalew LE, Tikoo SK, Zhou Y 2016 DDX3 interacts with influenza A virus NS1 and NP proteins and exerts antiviral function through regulation of stress granule formation. J Virol 90:3661–3675. doi:10.1128/JVI.03010-15. 26792746 PMC4794679

[39] Oh SW, Onomoto K, Wakimoto M, Onoguchi K, Ishidate F, Fujiwara T, Yoneyama M, Kato H, Fujita T 2016 Leader-containing uncapped viral transcript activates RIG-I in antiviral stress granules. PLoS Pathog 12:e1005444. doi:10.1371/journal.ppat.1005444. 26862753 PMC4749238

[40] Reineke LC, Kedersha N, Langereis MA, van Kuppeveld FJ, Lloyd RE 2015 Stress granules regulate double-stranded RNA-dependent protein kinase activation through a complex containing G3BP1 and Caprin1. mBio 6:e02486. doi:10.1128/mBio.02486-14. 25784705 PMC4453520

[41] Reineke LC, Lloyd RE 2015 The stress granule protein G3BP1 recruits protein kinase R to promote multiple innate immune antiviral responses. J Virol 89:2575–2589. doi:10.1128/JVI.02791-14. 25520508 PMC4325707

[42] White JP, Cardenas AM, Marissen WE, Lloyd RE 2007 Inhibition of cytoplasmic mRNA stress granule formation by a viral proteinase. Cell Host Microbe 2:295–305. doi:10.1016/j.chom.2007.08.006. 18005751

[43] Panas MD, Varjak M, Lulla A, Eng KE, Merits A, Karlsson Hedestam GB, McInerney GM 2012 Sequestration of G3BP coupled with efficient translation inhibits stress granules in Semliki Forest virus infection. Mol Biol Cell 23:4701–4712. doi:10.1091/mbc.E12-08-0619. 23087212 PMC3521679

[44] Silverman RH, Skehel JJ, James TC, Wreschner DH, Kerr IM 1983 rRNA cleavage as an index of ppp(A2’p)nA activity in interferon-treated encephalomyocarditis virus-infected cells. J Virol 46:1051–1055. doi:10.1128/JVI.46.3.1051-1055.1983. 6190010 PMC256583

[45] Dong B, Silverman RH 1995 2-5A-dependent RNase molecules dimerize during activation by 2-5A. J Biol Chem 270:4133–4137. doi:10.1074/jbc.270.8.4133. 7876164

[46] Malathi K, Dong B, Gale M Jr, Silverman RH 2007 Small self-RNA generated by RNase L amplifies antiviral innate immunity. Nature 448:816–819. doi:10.1038/nature06042. 17653195 PMC3638316

[47] Chakrabarti A, Banerjee S, Franchi L, Loo YM, Gale M Jr, Nunez G, Silverman RH 2015 RNase L activates the NLRP3 inflammasome during viral infections. Cell Host Microbe 17:466–477. doi:10.1016/j.chom.2015.02.010. 25816776 PMC4393362

[48] Siddiqui MA, Mukherjee S, Manivannan P, Malathi K 2015 RNase L cleavage products promote switch from autophagy to apoptosis by caspase-mediated cleavage of beclin-1. Int J Mol Sci 16:17611–17636. doi:10.3390/ijms160817611. 26263979 PMC4581211

[49] Manivannan P, Reddy V, Mukherjee S, Clark KN, Malathi K 2019 RNase L induces expression of a novel serine/threonine protein kinase, DRAK1, to promote apoptosis. Int J Mol Sci 20:E3535. doi:10.3390/ijms20143535. 31330998 PMC6679093

[50] Dong B, Niwa M, Walter P, Silverman RH 2001 Basis for regulated RNA cleavage by functional analysis of RNase L and Ire1p. RNA 7:361–373. doi:10.1017/s1355838201002230. 11333017 PMC1370093

[51] McCormick C, Khaperskyy DA 2017 Translation inhibition and stress granules in the antiviral immune response. Nat Rev Immunol 17:647–660. doi:10.1038/nri.2017.63. 28669985

[52] Wheeler JR, Jain S, Khong A, Parker R 2017 Isolation of yeast and mammalian stress granule cores. Methods 126:12–17. doi:10.1016/j.ymeth.2017.04.020. 28457979 PMC5924690

[53] Kim SS, Sze L, Liu C, Lam KP 2019 The stress granule protein G3BP1 binds viral dsRNA and RIG-I to enhance interferon-beta response. J Biol Chem 294:6430–6438. doi:10.1074/jbc.RA118.005868. 30804210 PMC6484135

[54] Yang W, Ru Y, Ren J, Bai J, Wei J, Fu S, Liu X, Li D, Zheng H 2019 G3BP1 inhibits RNA virus replication by positively regulating RIG-I-mediated cellular antiviral response. Cell Death Dis 10:946. doi:10.1038/s41419-019-2178-9. 31827077 PMC6906297

[55] Malathi K, Paranjape JM, Bulanova E, Shim M, Guenther-Johnson JM, Faber PW, Eling TE, Williams BR, Silverman RH 2005 A transcriptional signaling pathway in the IFN system mediated by 2′-5′-oligoadenylate activation of RNase L. Proc Natl Acad Sci U S A 102:14533–14538. doi:10.1073/pnas.0507551102. 16203993 PMC1239948

[56] Garcin D, Marq JB, Goodbourn S, Kolakofsky D 2003 The amino-terminal extensions of the longer Sendai virus C proteins modulate pY701-Stat1 and bulk Stat1 levels independently of interferon signaling. J Virol 77:2321–2329. doi:10.1128/jvi.77.4.2321-2329.2003. 12551969 PMC141115

[57] Strahle L, Garcin D, Le Mercier P, Schlaak JF, Kolakofsky D 2003 Sendai virus targets inflammatory responses, as well as the interferon-induced antiviral state, in a multifaceted manner. J Virol 77:7903–7913. doi:10.1128/jvi.77.14.7903-7913.2003. 12829830 PMC161935

[58] Oda K, Oda T, Matoba Y, Sato M, Irie T, Sakaguchi T 2017 Structural analysis of the STAT1:STAT2 heterodimer revealed the mechanism of Sendai virus C protein-mediated blockade of type 1 interferon signaling. J Biol Chem 292:19752–19766. doi:10.1074/jbc.M117.786285. 28978648 PMC5712616

[59] Sakaguchi T, Kato A, Kiyotani K, Yoshida T, Nagai Y 2008 Studies on the paramyxovirus accessory genes by reverse genetics in the Sendai virus-mouse system. Proc Jpn Acad Ser B Phys Biol Sci 84:439–451. doi:10.2183/pjab.84.439. PMC3720547 19075516

[60] Chakrabarti A, Jha BK, Silverman RH 2011 New insights into the role of RNase L in innate immunity. J Interferon Cytokine Res 31:49–57. doi:10.1089/jir.2010.0120. 21190483 PMC3021357

[61] Chakrabarti A, Ghosh PK, Banerjee S, Gaughan C, Silverman RH 2012 RNase L triggers autophagy in response to viral infections. J Virol 86:11311–11321. doi:10.1128/JVI.00270-12. 22875977 PMC3457150

[62] Siddiqui MA, Malathi K 2012 RNase L induces autophagy via c-Jun N-terminal kinase and double-stranded RNA-dependent protein kinase signaling pathways. J Biol Chem 287:43651–43664. doi:10.1074/jbc.M112.399964. 23109342 PMC3527951

[63] Aulas A, Fay MM, Lyons SM, Achorn CA, Kedersha N, Anderson P, Ivanov P 2017 Stress-specific differences in assembly and composition of stress granules and related foci. J Cell Sci 130:927–937. doi:10.1242/jcs.199240. 28096475 PMC5358336

[64] Emara MM, Fujimura K, Sciaranghella D, Ivanova V, Ivanov P, Anderson P 2012 Hydrogen peroxide induces stress granule formation independent of eIF2alpha phosphorylation. Biochem Biophys Res Commun 423:763–769. doi:10.1016/j.bbrc.2012.06.033. 22705549 PMC3399031

[65] Sun Y, Dong L, Yu S, Wang X, Zheng H, Zhang P, Meng C, Zhan Y, Tan L, Song C, Qiu X, Wang G, Liao Y, Ding C 2017 Newcastle disease virus induces stable formation of bona fide stress granules to facilitate viral replication through manipulating host protein translation. FASEB J 31:1337–1353. doi:10.1096/fj.201600980R. 28011649

[66] Berlanga JJ, Ventoso I, Harding HP, Deng J, Ron D, Sonenberg N, Carrasco L, de Haro C 2006 Antiviral effect of the mammalian translation initiation factor 2alpha kinase GCN2 against RNA viruses. EMBO J 25:1730–1740. doi:10.1038/sj.emboj.7601073. 16601681 PMC1440839

[67] Zhang P, Li Y, Xia J, He J, Pu J, Xie J, Wu S, Feng L, Huang X, Zhang P 2014 IPS-1 plays an essential role in dsRNA-induced stress granule formation by interacting with PKR and promoting its activation. J Cell Sci 127:2471–2482. doi:10.1242/jcs.139626. 24659800

[68] Burke JM, Lester ET, Tauber D, Parker R 2020 RNase L promotes the formation of unique ribonucleoprotein granules distinct from stress granules. J Biol Chem 295:1426–1438. doi:10.1074/jbc.RA119.011638. 31896577 PMC7008361

[69] Li Y, Banerjee S, Wang Y, Goldstein SA, Dong B, Gaughan C, Silverman RH, Weiss SR 2016 Activation of RNase L is dependent on OAS3 expression during infection with diverse human viruses. Proc Natl Acad Sci U S A 113:2241–2246. doi:10.1073/pnas.1519657113. 26858407 PMC4776461

[70] Burke JM, Moon SL, Matheny T, Parker R 2019 RNase L reprograms translation by widespread mRNA turnover escaped by antiviral mRNAs. Mol Cell 75:1203–1217 e5. doi:10.1016/j.molcel.2019.07.029. 31494035 PMC6754297

[71] Rath S, Prangley E, Donovan J, Demarest K, Wingreen NS, Meir Y, Korennykh A 2019 Concerted 2-5A-mediated mRNA decay and transcription reprogram protein synthesis in the dsRNA response. Mol Cell 75:1218–1228 e6. doi:10.1016/j.molcel.2019.07.027. 31494033 PMC6754276

[72] Cirillo L, Cieren A, Barbieri S, Khong A, Schwager F, Parker R, Gotta M 2020 UBAP2L forms distinct cores that act in nucleating stress granules upstream of G3BP1. Curr Biol 30:698–707.e6. doi:10.1016/j.cub.2019.12.020. 31956030

[73] Saito T, Hirai R, Loo YM, Owen D, Johnson CL, Sinha SC, Akira S, Fujita T, Gale M Jr 2007 Regulation of innate antiviral defenses through a shared repressor domain in RIG-I and LGP2. Proc Natl Acad Sci U S A 104:582–587. doi:10.1073/pnas.0606699104. 17190814 PMC1766428

[74] Mercado-López X, Cotter CR, Kim W-K, Sun Y, Muñoz L, Tapia K, López CB 2013 Highly immunostimulatory RNA derived from a Sendai virus defective viral genome. Vaccine 31:5713–5721. doi:10.1016/j.vaccine.2013.09.040. 24099876 PMC4406099

[75] Xu J, Mercado-López X, Grier JT, Kim W-K, Chun LF, Irvine EB, Del Toro Duany Y, Kell A, Hur S, Gale M, Raj A, López CB 2015 Identification of a natural viral RNA motif that optimizes sensing of viral RNA by RIG-I. mBio 6:e01265-15. doi:10.1128/mBio.01265-15. 26443454 PMC4611036

[76] Yoshida A, Kawabata R, Honda T, Tomonaga K, Sakaguchi T, Irie T 2015 IFN-beta-inducing, unusual viral RNA species produced by paramyxovirus infection accumulated into distinct cytoplasmic structures in an RNA-type-dependent manner. Front Microbiol 6:804. 26300870 10.3389/fmicb.2015.00804 PMC4523817

[77] Malathi K, Saito T, Crochet N, Barton DJ, Gale M Jr, Silverman RH 2010 RNase L releases a small RNA from HCV RNA that refolds into a potent PAMP. RNA 16:2108–2119. doi:10.1261/rna.2244210. 20833746 PMC2957051

[78] Hartmann R, Justesen J, Sarkar SN, Sen GC, Yee VC 2003 Crystal structure of the 2′-specific and double-stranded RNA-activated interferon-induced antiviral protein 2′-5′-oligoadenylate synthetase. Mol Cell 12:1173–1185. doi:10.1016/s1097-2765(03)00433-7. 14636576

[79] Sumpter R Jr, Loo YM, Foy E, Li K, Yoneyama M, Fujita T, Lemon SM, Gale M Jr 2005 Regulating intracellular antiviral defense and permissiveness to hepatitis C virus RNA replication through a cellular RNA helicase, RIG-I. J Virol 79:2689–2699. doi:10.1128/JVI.79.5.2689-2699.2005. 15708988 PMC548482

[80] Qi LS, Larson MH, Gilbert LA, Doudna JA, Weissman JS, Arkin AP, Lim WA 2013 Repurposing CRISPR as an RNA-guided platform for sequence-specific control of gene expression. Cell 152:1173–1183. doi:10.1016/j.cell.2013.02.022. 23452860 PMC3664290

[81] Mali P, Yang L, Esvelt KM, Aach J, Guell M, DiCarlo JE, Norville JE, Church GM 2013 RNA-guided human genome engineering via Cas9. Science 339:823–826. doi:10.1126/science.1232033. 23287722 PMC3712628

[82] Aulas A, Fay MM, Szaflarski W, Kedersha N, Anderson P, Ivanov P 2017 Methods to classify cytoplasmic foci as mammalian stress granules. J Vis Exp 123:e55656. doi:10.3791/55656. PMC5607937 28570526

[83] Thakur CS, Jha BK, Dong B, Das Gupta J, Silverman KM, Mao H, Sawai H, Nakamura AO, Banerjee AK, Gudkov A, Silverman RH 2007 Small-molecule activators of RNase L with broad-spectrum antiviral activity. Proc Natl Acad Sci U S A 104:9585–9590. doi:10.1073/pnas.0700590104. 17535916 PMC1877983

[84] Dayal S, Zhou J, Manivannan P, Siddiqui MA, Ahmad OF, Clark M, Awadia S, Garcia-Mata R, Shemshedini L, Malathi K 2017 RNase L suppresses androgen receptor signaling, cell migration and matrix metalloproteinase activity in prostate cancer cells. Int J Mol Sci 18:E529. doi:10.3390/ijms18030529. 28257035 PMC5372545

